# Design, Synthesis, and Cytotoxic Activity of Novel Natural Arylsulfonamide-Inspired Molecules

**DOI:** 10.3390/molecules27051479

**Published:** 2022-02-22

**Authors:** Wenbo Huang, Liqiao Shi, Manli Liu, Zhigang Zhang, Fang Liu, Tong Long, Shaohua Wen, Daye Huang, Kaimei Wang, Ronghua Zhou, Wei Fang, Hongtao Hu, Shaoyong Ke

**Affiliations:** 1 Key Laboratory of Microbial Pesticides, Ministry of Agriculture and Rural Affairs, Wuhan 430064, China; huangwenbo@nberc.com (W.H.); liqiao.shi@nberc.com (L.S.); manli.liu@nberc.com (M.L.); zhigang.zhang@nberc.com (Z.Z.); fang.liu@nberc.com (F.L.); tong.long@nberc.com (T.L.); wenshaohua1985@163.com (S.W.); xiaohuangdaye@126.com (D.H.); kaimei.wang@nberc.com (K.W.); ronghua.zhou@nberc.com (R.Z.); 2 National Biopesticide Engineering Research Centre, Hubei Biopesticide Engineering Research Centre, Hubei Academy of Agricultural Sciences, Wuhan 430064, China

**Keywords:** natural arylsulfonamides, derivatives, synthesis, biological evaluation, SARs

## Abstract

Primary arylsulfonamide functional groups feature prominently in diverse pharmaceuticals. However, natural arylsulfonamides are relatively infrequent. In this work, two novel arylsulfonamide natural products were first synthesized, and then a series of novel molecules derived from natural arylsulfonamides were designed and synthesized, and their in vitro cytotoxic activities against A875, HepG2, and MARC145 cell lines were systematically evaluated. The results indicate that some of these arylsulfonamide derivatives exhibit significantly good cytotoxic activity against the tested cell lines compared with the control 5-fluorouracil (5-FU), such as compounds **10l**, **10p**, **10q**, and **10r**. In particular, the potential molecule **10q**, containing a carbazole moiety, exhibited the highest inhibitory activity against all tested cell lines, with IC_50_ values of 4.19 ± 0.78, 3.55 ± 0.63, and 2.95 ± 0.78 μg/mL, respectively. This will offer the potential to discover novel drug-like compounds from the sparsely populated area of natural products that can lead to effective anticancer agents.

## 1. Introduction

Natural products (NPs) and their molecular scaffolds have a long tradition of offering chemists a range of uncharted chemotypes as valuable starting points for the development of novel drugs [1,2,3], and have also been a major source of anticancer drug discovery [4,5,6,7].

**Figure 1:**
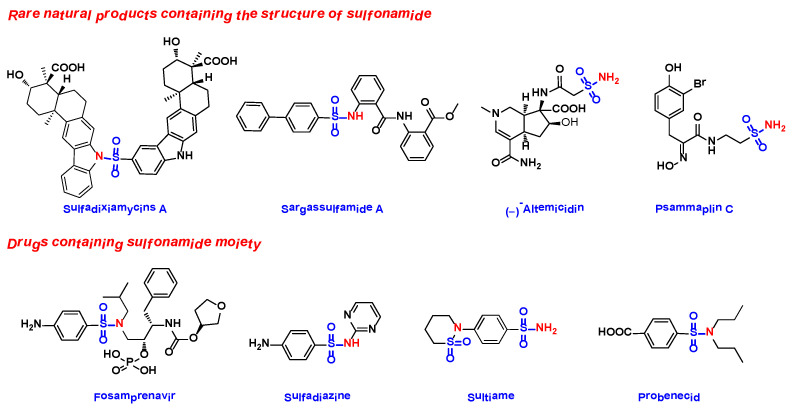
Prototypical natural products and drugs containing a sulfonamide group.

Sulfonamides have a functional group (R-SO_2_-NH_2_) called the sulfonamide group; sulfonamide scaffolds are also very important structural motifs that are present in diverse pharmaceuticals (Figure 1), showing important and extensive biological activities, such as antitumor [8,9,10,11,12], anti-inflammatory [13,14], and antiviral activities [15,16]. However, due to the structural particularity, natural products that contain a primary sulfonamide group in their structures are rare. Sulfadixiamycin A, (−)-altemicidin, and psammaplin C are a few examples of natural products that contain the structure of a sulfonamide moiety [17,18] (Figure 1).

Recently, sargassulfamide A, bearing a rare and unique sulfonamide group, was isolated from the seaweed *Sargassum naozhouense* [19]. Almost simultaneously, another novel arylsulfonamide natural product was isolated together with sargassulfamide A from the roots and rhizomes of *Tupistra chinensis* Baker [20], whereas the result of in vitro cytotoxic activity in the human cancer cell lines indicated that the novel arylsulfonamide natural products displayed weak cytotoxicity.

Natural products are among the most important sources of the lead compounds in drug discovery; however, most of them cannot be used as drugs directly. In addition, owing to their low fermentation and extraction levels, further research exploring the potential bioactivity of some natural compounds has been seriously limited. Accordingly, structural optimization and derivation of natural products becomes a powerful strategy for improving the efficiency and success rate of NP-based drug development. Our research group has committed to discovering highly active compounds from natural products [21,22,23,24,25,26], so the rare natural product sargassulfamide A, bearing a privileged scaffold of arylsulfonamide, aroused our interest. It is well known that molecular skeletons determine whether molecules have bioactivity; despite the natural arylsulfonamide sargassulfamide A being less effective against the human cancer cell lines, we wondered whether we could modulate or improve the activities of these analogues of natural products by changing the position of the amide unit and pharmacophore hybridization.

**Figure 2:**
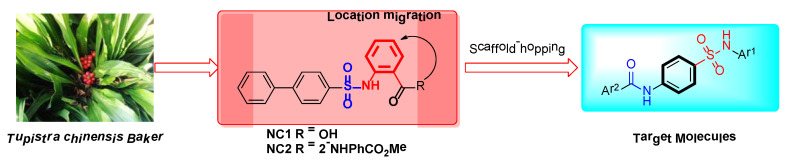
Design strategy of natural arylsulfonamide-inspired molecules.

Meanwhile, aryls and hetero-aryls are important pharmacological units that always increase the potential bioactivity of corresponding molecules [27,28,29]. Hence, several different types of (hetero)aryl groups—including biphenyl, diphenyl ether, and heterocycles—were selected to construct diversity molecules, and theses selected aromatic aryl amines (Ar^1^NH_2_) and aryl acids (Ar^2^COOH) are usually classical pharmacological units. Taking into account the above considerations, we wish herein to report the molecule design, convenient synthesis, and biological evaluation of a series of novel natural arylsulfonamide-inspired molecules (Figure 2).

**Scheme 1:**
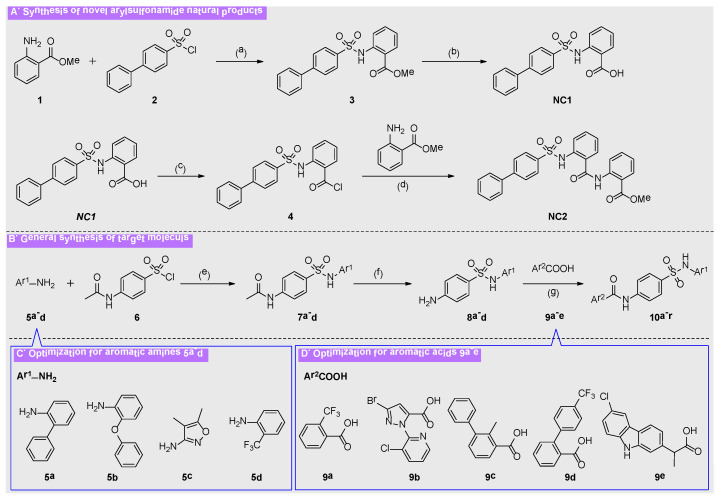
Synthesis of natural arylsulfonamides and aryl-carboxamide derivatives bearing sulfonamide units **10a**–**r**. Reagents and conditions: a. Et_3_N, DCM, rt; b. NaOH, MeOH/H_2_O, 60 °C; c. SOCl_2_, reflux; d. Et_3_N, DCM, rt; e. Et_3_N, DCM, rt; f. NaOH, MeOH/H_2_O, 45-50 °C; g. Ar^2^COOH, HOBt, EDCI, Et_3_N, DMF, r.t.

In this study, sargassulfamide A (**NC2**) was used as a key starting point for the development of high-efficiency cytotoxic agents; a series of novel derivatives containing arylsulfonamide and carboxamide pharmacophores **10a**-**r** were designed and synthesized as shown in Scheme 1, and their cytotoxic activities against several human cancer cell lines—including A875, HepG2, and MARC145—were systematically evaluated by the MTT colorimetric method for the first time. Beyond that, the possible structure and activity relationships are also summarized and discussed.

## 2. Results and Discussion

Chemistry: In this work, a series of novel aryl-carboxamide derivatives bearing sulfonamide units were designed and synthesized by integrating natural arylsulfonamides with carboxamide pharmacophores. The general synthetic route for these compounds is outlined in Scheme 1.

Synthesis of natural arylsulfonamide-containing carboxylic acid (**NC1**) and sargassulfamide A (**NC2**): First, the ethyl anthranilate **1** was treated with the biphenylsulfonyl chloride **2** via sulfonamidation reaction to yield the key intermediate arylsulfonamide **3**; subsequently, compound **3** was conveniently hydrolyzed to the natural compound **NC1**, which was transformed to the corresponding acyl chloride **4** and then reacted with the ethyl anthranilate to obtain the target sargassulfamide A (**NC2**).

Synthesis of aryl-carboxamide derivatives bearing sulfonamide units **10a–r**: The arylsulfonamide derivatives **7a**–**d** were prepared *via* the reaction of various aryl amines and 4-acetamidobenzene-1-sulfonyl chloride **6** in the presence of Et_3_N/DCM, and then the corresponding arylsulfonamide derivatives were transformed into the intermediate arylsulfonamide derivatives **8a**–**d** via deacetylation reaction. Subsequently, the obtained compounds **8a**–**d** were condensed with various aromatic acids **9a**–**e** under EDCI/HOBt-promoted conditions to construct target aryl-carboxamide derivatives bearing sulfonamide units **10a**–**r**.

**Figure 3:**
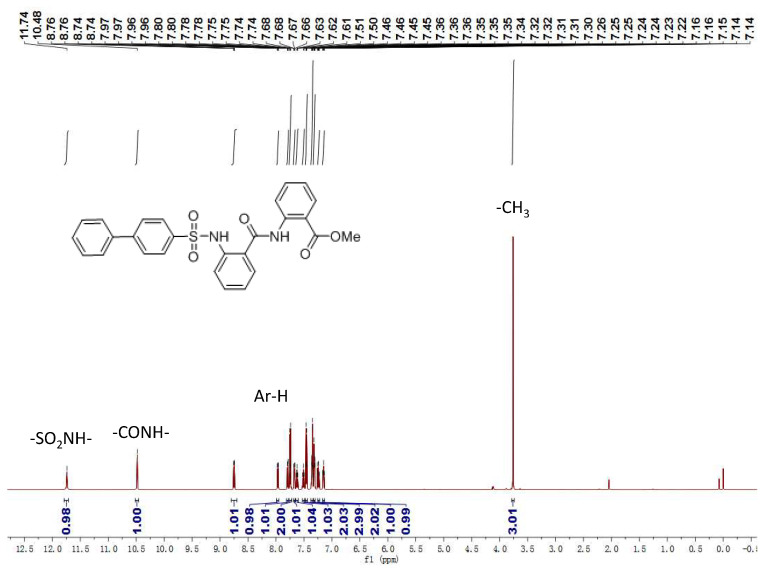
Representative ^1^H NMR spectral analysis of sargassulfamide A.

Spectroscopy studies: The structures of all of the target compounds in this work gave satisfactory chemical analyses, including ^1^H NMR, ^13^C NMR, ^19^F NMR, and mass spectroscopy, and all of these spectral data were in good agreement with the proposed structures. For the ^1^H NMR spectrum of all arylsulfonamide derivatives, the signals at 11.74–10.76 ppm showed a single peak, which was attributed to NH protons attached to the sulfonyl group as indicated in the molecular structures. Correspondingly, the signals in the range of 10.65–9.40 ppm were assigned to the NH protons, which attached to the carbonyl group. The signal peaks of the remaining at low fields were attributed to aromatic ring proton peaks. All of the characteristic peaks observed within the ^1^H NMR spectra for title compounds are given in the Experimental section, and the representative ^1^H NMR spectral analysis of the target sargassulfamide A is shown in Figure 3.

**Figure 4:**
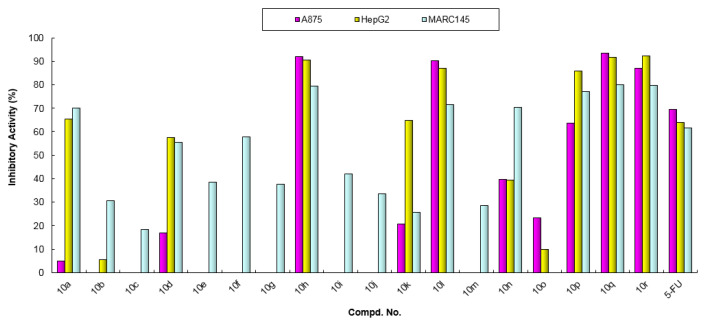
Inhibitory activity of compounds **10a**–**r** at the concentration of 40 µg/mL. Abbreviations: A875—human melanoma cell line; HepG2—human hepatocellular liver carcinoma cell line; MARC145—a subclone of African green monkey kidney cell line MA-104; 5-FU—5-fluorouracil, used as a positive control.

Pharmacological evaluation: All target carboxamide derivatives derived from natural arylsulfonamides **10a**–**r** were screened for their potential in vitro cytotoxic effects on A875 (human melanoma), HepG2 (human hepatocellular liver carcinoma), and MARC145 (A subclone of African green monkey kidney cell line MA-104) cell lines using the standard MTT assay [30,31], with 5-FU (5-fluorouracil) as a positive control. The results are summarized in Figure 4 and Table 1. The IC_50_ value represents the drug concentration required to inhibit cell growth by 50%.

Based on the preliminary screening results, we found that some of these arylsulfonamide derivatives indicated moderate-to-good inhibition activity against all tested cell lines. Notably, the compounds **10l**, **10p**, **10q**, and **10r** exhibited significantly higher activities compared with the control at a concentration of 40 µg/mL (Figure 4), demonstrating that these natural-product-inspired molecules derived from sargassulfamide A could be considered as promising scaffolds for developing effective anticancer agents.

In order to further explore their potential antitumor activity, the IC_50_ values of two novel arylsulfonamide natural products and a series of novel molecules derived from natural arylsulfonamide derivatives were all systematically evaluated. The potential inhibitory activities expressed as IC_50_ values for all compounds are shown in Table 1. As indicated in Table 1, the natural arylsulfonamide **NC1** showed poor activity against tested cell lines, and sargassulfamide A (**NC2**) almost completely lost inhibitory activity (IC_50_ > 40 µg/mL). Compounds **10l**, **10p 10q**, and **10r**, containing a trifluoromethyl unit, had higher inhibitory effects on all cell lines compared to the positive control 5-FU. It should be noted that introduction of a trifluoromethyl group to natural products and organic molecules is vitally important for lipophilicity, hydrophobicity, and metabolic stability. Hence, trifluoromethylation has played an important role in discovering high-activity drugs [32]. Compound **10q**, containing a carbazole scaffold, showed the strongest inhibitory effect on all three cell lines, with an IC_50_ of 4.19 ± 0.78 (A875), 3.55 ± 0.63 (HepG2), and 2.95 ± 0.78 (MARC145) μg/mL, which may be due to the carbazole ring being favorable for such activity. The results showed in Table 1 can further demonstrate the cytotoxic effect of arylsulfonamide derivatives as potential anticancer reagents for cancer cell lines.

**Figure 5:**
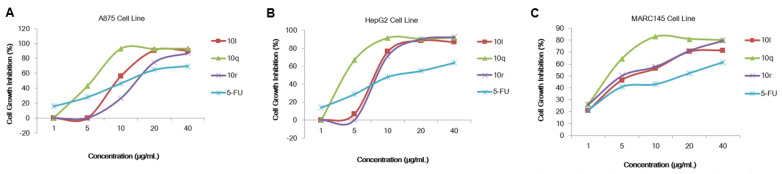
Dose–response analysis of cell growth inhibition activity for compounds **10l**, **10q**, **10r**, and **5-FU** (positive control) against A875 cells (**A**), HepG2 cells (**B**), and MARC145 cells (**C**).

Subsequently, the dose–response analysis for the high-potential compounds **10l**, **10q**, **10r**, and 5-FU was conducted, as shown in Figure 5, indicating that the cytotoxic effects of arylsulfonamide derivatives on A875 cells (left), HepG2 cells (middle), and MARC145 cells (right) are significantly concentration-dependent.

**Figure 6:**
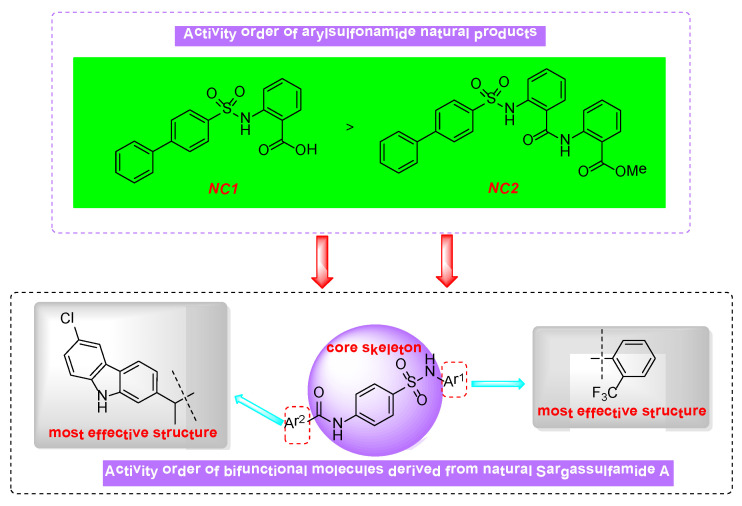
General SARs of target compounds.

Structure and activity relationships (SARs): The structural evolution here was to modify natural arylsulfonamides with diverse aryl amines (**5a**–**d**) and aromatic acid scaffolds (**9a**–**e**). According to the in vitro bioassay results presented in Figure 4 and Table 1, we obtained the preliminary structure–activity relationships (SARs) for these prepared arylsulfonamides derivatives. We concluded that the results indicate that modifying the carboxyl group of natural arylsulfonamide can yield superior cytotoxicity activity against different cancer cells. Meanwhile, changing the position of the amide unit also significantly affected the antitumor activity (Figure 6). In addition, the compounds containing trifluoromethyl-substituted arylsulfonamide derivatives presented greater cytotoxicity activity (**10d**, **10h**, **10l**, and **10p**). In particular, the compounds containing a special carbazole moiety showed the highest inhibitory activity (**10q** and **10r**), indicating that the carbazole ring is beneficial to increase the activity. It should be noted that carbazole skeletons are ubiquitous in diverse antitumor pharmaceuticals [33,34,35]. Above all, the obtained SARs could contribute to the discovery of potential cytotoxic agents from natural products in our subsequent research.

## 3. Conclusions

In summary, we have described the molecular design, synthesis, and biological evaluation of novel arylsulfonamides inspired by natural sulfadixiamycin A. These novel natural arylsulfonamides and their derivatives were conveniently synthesized and characterized by typical spectral analyses, including ^1^H NMR, ^13^C NMR, ^19^F NMR, and ESI-MS. The preliminary in vitro bioassay indicated that some of these arylsulfonamides bearing carbazole rings displayed good cytotoxic activity against the A875 and HepG2 cancer cell lines, and the high-potential molecule **10q** exhibited the highest inhibitory activity against all tested cell lines, with IC_50_ values of 4.19 ± 0.78, 3.55 ± 0.63, and 2.95 ± 0.78 μg/mL, respectively. However, the possible mechanisms for their antitumor activities still need to be explored further, and these results may promote further development of our research inspired by natural products to discover potential drug candidates.

## 4. Experimental

Instrumentation and chemicals: All reagents from commercial sources were used directly, without purification. All melting points (m.p.) were measured using a digital model X-5 apparatus (Shanghai Instrument Physical Optics Instrument Co., LTD, Shanghai, China), and were uncorrected. ^1^H NMR, ^13^C NMR, and ^19^F NMR spectra were recorded on a Bruker Avance III 600 MHz FT-NMR spectrometer (Bruker, Billerica, MA, USA), using CDCl_3_ or DMSO-*d*_6_ as the solvent and tetramethylsilane (TMS) as the internal standard. Chemical shifts are reported in *δ* (parts per million) values, and coupling constants ^n^*J* are reported in Hz. Mass spectra were recorded on a Waters ACQUITY UPLC^®^ H-Class PDA (Waters^®^) instrument (Waters^®^, Milford, MA, USA). Thin-layer chromatography (TLC) was carried out on precoated GF254 silica gel plates (Qingdao Haiyang Chemical, Qingdao, China), and spots were visualized with ultraviolet light. All commercially available starting materials and reagents were used without further purification, unless otherwise specified.

Synthetic procedures for natural compound **NC1**: To a solution of ethyl anthranilate **1** (3.30 g, 20 mmol) and triethylamine (4.04 g, 40 mmol) in anhydrous DCM (40 mL), we added 4-biphenylsulfonyl chloride (6.06 g, 24 mmol) dropwise, which dissolved in DCM under an ice bath, and then the mixture was stirred at room temperature overnight and detected by thin-layer chromatography. After the completion of the reaction, we washed the crude mixture with saturated aqueous NaHCO_3_ and brine solution, and then the mixture was extracted with DCM and dried over Na_2_SO_4_. The obtained crude product **3** was used for the next reaction without further purification. To the obtained compound **3** in methanol (80 mL), we added 20 mL of aqueous sodium hydroxide (0.1 mol), which was stirred at room temperature overnight. After the completion of the reaction, the mixture was adjusted to pH 3 with diluted hydrochloric acid under an ice bath, and the precipitate was filtered and washed with a small amount of alcohol to yield the pure natural compound **NC1**. The physico-chemical properties for compound **NC1** are as follows, and the corresponding spectrum are indicated in Supplementary Materials.

*2-([1,1′-Biphenyl]-4-sulfonamido)benzoic acid* (**NC1**), white solid, m.p. 205–207 °C. ^1^H NMR (600 MHz, DMSO-d*_6_*, 25 °C) *δ* = 11.22 (s, 1H), 7.94–7.89 (m, 3H), 7.86 (dd, *J* = 8.4, 1.4 Hz, 2H), 7.71–7.69 (m, 2H), 7.59–7.56 (m, 2H), 7.50–7.48 (m, 2H), 7.46–7.42 (m, 1H), 7.16–7.12 (m, 1H) ppm. ^13^C NMR (151 MHz, DMSO-*d_6_*, 25 °C) *δ* = 170.3, 145.4, 140.2, 138.5, 137.8, 135.1, 132.1, 129.6, 129.2, 128.1, 128.0, 127.6, 123.8, 118.8, 117.1 ppm. MS *m*/*z* 354.2 = [M + H]^+^, calculated for C_19_H_15_NO_4_S *m*/*z* = 353.0.

Synthetic procedures for natural compound **NC2**: Thionyl chloride (25 mL) was added dropwise under an ice bath to natural compound **NC1** (1.59 g, 4.5 mmol); after addition, the mixture was slowly heated to 80 °C overnight, and then the reaction solution was concentrated to obtain compound **4**, which was used for the next reaction without further purification. To a mixture of ethyl anthranilate (0.83 g, 5 mmol) and triethylamine (1.2 equivalents) in CH_2_Cl_2_ (25 mL), we added compound **4** dropwise under an ice bath, and the resulting mixture was stirred at room temperature until TLC indicated that the reaction was complete. Then, 30 mL of water was added to the mixture, the aqueous solution was extracted with CH_2_Cl_2_, and the combined organic phases were washed with NaHCO_3_ and dried with anhydrous Na_2_SO_4_. After removing the volatile component, the desired natural compound **NC2** was obtained. Sargassulfamide A (**NC2**) was subjected to isolation with silica column chromatography (eluting solution: petroleum ether/ethyl acetate = 3/1 (*v*/*v*)). The physico-chemical properties for compound **NC2** are as follows, and the corresponding spectrum are indicated in Supplementary Materials.

*Methyl 2-(2-([1,1′-biphenyl]-4-sulfonamido)benzamido)benzoate* (**NC2**), white solid, m.p. 148–149 °C. ^1^H NMR (600 MHz, DMSO-d*_6_*, 25 °C) *δ* = 11.74 (s, 1H), 10.48 (s, 1H), 8.75 (dd, *J* = 8.4, 0.7 Hz, 1H), 7.97 (dd, *J* = 8.0, 1.6 Hz, 1H), 7.79 (dd, *J* = 8.3, 0.9 Hz, 1H), 7.76–7.72 (m, 2H), 7.67 (dd, *J* = 7.9, 1.3 Hz, 1H), 7.65–7.60 (m, 1H), 7.53–7.49 (m, 1H), 7.47–7.44 (m, 2H), 7.37–7.34 (m, 3H), 7.33–7.30 (m, 2H), 7.25–7.22 (m, 1H), 7.17–7.13 (m, 1H), 3.76 (s, 3H) ppm. ^13^C NMR (151 MHz, DMSO-*d_6_*, 25 °C) *δ* = 168.0, 166.6, 144.9, 139.6, 138.3, 137.6, 137.2, 134.6, 133.3, 131.0, 129.5, 129.1, 128.7, 128.0, 127.7, 127.3, 126.3, 125.9, 124.5, 123.7, 122.1, 118.6, 53.0 ppm. MS *m*/*z* 487.4 = [M + H]^+^, calculated for C_27_H_22_N_2_O_5_S *m*/*z* = 486.1.

General synthetic procedures for aryl-carboxamide derivatives bearing sulfonamide units **10a**–**r**: To a solution of aryl amine (10 mmol) and triethylamine (12 mmol, 1.2 equiv.) in anhydrous DCM (20 mL), we added phenylsulfonyl chloride (12 mmol) dropwise, which dissolved in DCM under an ice bath. After the completion of the reaction, we washed the crude mixture with saturated aqueous NaHCO_3_ and brine solution, and then the mixture was extracted with DCM and dried over Na_2_SO_4_. The obtained crude products **7a**–**d** were used for the next reaction without further purification. The crude products **7a**–**d** (1 equivalent) in methanol (20 mL) was added to 5 mL of aqueous sodium hydroxide (4 equivalents), and then the mixture stirred at 50 °C for 10 h. After the completion of the reaction, the mixture was adjusted to pH 5–6 with diluted hydrochloric acid under an ice bath, and the precipitate was filtered and washed with a small amount of alcohol to yield **8a**–**d**. To a solution of **8a**–**d** (1.2 mmol) in DMF (4 mL), we added HOBt (1.5 mmol), EDCI (1.5 mmol), Et_3_N (2.5 mmol) and appropriate substituted acids **9a**–**e** (1.0 mmol), and then the mixture was stirred at room temperature overnight. After the completion of the reaction, water was added to the mixture, which was extracted with DCM, and the organic layer was washed with water and brine, and then dried with anhydrous Na_2_SO_4_. The solvent was removed to yield crude target compounds, which were purified by silica gel column chromatography (eluting solution: petroleum ether/ethyl acetate = 2/1 (*v*/*v*)) or recrystallization to yield pure compounds. The physico-chemical properties for some intermediates and target compounds are as follows, and the corresponding spectrum are indicated in Supplementary Materials.

*N-([1,1′-Biphenyl]-2-yl)-4-aminobenzenesulfonamide* (**8a**), m.p. 128–129 °C. MS *m/z* 347.3 = [M + Na]^+^, calculated for C_18_H_16_N_2_O_2_S *m*/*z* = 324.1.

*4-Amino-N-(2-phenoxyphenyl)benzenesulfonamide* (**8b**), m.p. 119–120 °C. MS *m*/*z* 363.4 = [M + Na]^+^, calculated for C_18_H_16_N_2_O_3_S *m/z* = 340.1.

*4-Amino-N-(4,5-dimethylisoxazol-3-yl)benzenesulfonamide* (**8c**), m.p. 162–163 °C. MS *m*/*z* 290.3 = [M + Na]^+^, calculated for C_11_H_13_N_3_O_3_S *m/z* = 267.1.

*4-Amino-N-(2-(trifluoromethyl)phenyl)benzenesulfonamide* (**8d**), m.p. 95–96 °C. MS *m*/*z* 317.4 = [M + H]^+^, calculated for C_13_H_11_F_3_N_2_O_2_S *m/z* = 316.0.

*N-(4-(N-([1,1′-Biphenyl]-2-yl)sulfamoyl)phenyl)-2-(trifluoromethyl)benzamide* (**10a**), white solid, m.p. 151–152 °C. ^1^H NMR (600 MHz, CDCl_3,_ TMS, 25 °C) *δ* = 7.93 (s, 1H), 7.76 (d, *J* = 7.8 Hz, 1H), 7.69 (d, *J* = 8.2 Hz, 1H), 7.67–7.64 (m, 2H), 7.62 (d, *J* = 8.6 Hz, 3H), 7.50 (d, *J* = 8.7 Hz, 2H), 7.36 (dd, *J* = 5.1, 1.8 Hz, 4H), 7.18 (td, *J* = 7.5, 0.9 Hz, 1H), 7.12 (dd, *J* = 7.6, 1.5 Hz, 1H), 6.88 (dt, *J* = 7.5, 3.8 Hz, 2H), 6.65 (s, 1H) ppm. ^13^C NMR (151 MHz, DMSO-*d_6_*, 25 °C) *δ* = 165.9, 141.7, 137.2, 135.1, 134.5, 134.4, 133.4, 132.4, 130.7, 130.4, 129.2, 128.8, 128.7, 128.5, 128.4, 128.2, 127.2, 126.7, 125.3, 123.4 (q, *J* = 274.8 Hz), 122.0, 119.6. ^19^F NMR (565 MHz, DMSO-*d_6_*, 25 °C) *δ* = −58.8. MS *m*/*z* 497.3 = [M + H]^+^, calculated for C_26_H_19_F_3_N_2_O_3_S *m*/*z* = 496.1.

*N-(4-(N-(2-Phenoxyphenyl)sulfamoyl)phenyl)-2-(trifluoromethyl)benzamide* (**10b**), white solid, m.p. 161–162 °C. ^1^H NMR (600 MHz, DMSO-d*_6_*, 25 ^o^C) *δ* = 10.93 (s, 1H), 9.85 (s, 1H), 7.88 (d, *J* = 7.8 Hz, 1H), 7.82 (t, *J* = 7.5 Hz, 1H), 7.74 (t, *J* = 8.1 Hz, 4H), 7.70–7.66 (m, 2H), 7.42 (d, *J* = 7.5 Hz, 1H), 7.28 (t, *J* = 7.8 Hz, 2H), 7.12–7.07 (m, 3H), 6.71 (dd, *J* = 7.6, 1.5 Hz, 1H), 6.64 (d, *J* = 8.4 Hz, 2H) ppm. ^13^C NMR (151 MHz, DMSO-*d_6_*, 25 °C) *δ* = 171.2, 161.3, 154.8, 147.8, 140.9, 140.1, 138.0, 135.6, 134.9, 134.8, 133.8, 133.1, 133.0, 131.7, 131.2, 131.0, 129.0 (q, *J* = 273.3 Hz), 128.8, 128.6, 124.5, 123.7, 123.6. ^19^F NMR (565 MHz, DMSO-*d_6_*, 25 °C) *δ* = −53.1. MS *m*/*z* 513.5 = [M + H]^+^, calculated for C_26_H_19_F_3_N_2_O_4_S *m*/*z* = 512.1.

*N-(4-(N-(4,5-Dimethylisoxazol-3-yl)sulfamoyl)phenyl)-2-(trifluoromethyl)benzamide* (**10c**), white solid, m.p. 183–184 °C. ^1^H NMR (600 MHz, DMSO-d*_6_*, 25 ^o^C) *δ* = 10.99 (s, 1H), 10.65 (s, 1H), 7.89–7.84 (m, 5H), 7.82 (t, *J* = 7.5 Hz, 1H), 7.74 (t, *J* = 7.5 Hz, 2H), 2.23 (s, 3H), 1.80 (s, 3H) ppm. ^13^C NMR (151 MHz, DMSO-*d_6_*, 25 °C) *δ* = 166.6, 166.2, 157.2, 143.3, 136.1, 134.8, 133.2, 130.9, 129.0, 128.9, 127.0, 126.4, 124.2 (q, *J* = 273.3 Hz), 119.7, 105.0, 11.2, 6.4. ^19^F NMR (565 MHz, DMSO-*d_6_*, 25 °C) *δ* = −57.9. MS *m*/*z* 440.3 = [M + H]^+^, calculated for C_19_H_16_F_3_N_3_O_4_S *m/z* = 439.0.

*2-(Trifluoromethyl)-N-(4-(N-(2-(trifluoromethyl)phenyl)sulfamoyl)phenyl)benzamide* (**10d**), white solid, m.p. 128–129 °C. ^1^H NMR (600 MHz, DMSO-d*_6_*, 25 °C) *δ* = 10.91 (s, 1H), 9.92 (s, 1H), 8.54 (dd, *J* = 4.7, 1.5 Hz, 1H), 8.25 (dd, *J* = 8.1, 1.5 Hz, 1H), 7.83 (d, *J* = 8.7 Hz, 2H), 7.75 (d, *J* = 8.8 Hz, 2H), 7.71 (dd, *J* = 7.9, 1.1 Hz, 1H), 7.67 (dd, *J* = 8.1, 4.7 Hz, 1H), 7.58 (t, *J* = 7.3 Hz, 1H), 7.52 (s, 1H), 7.45 (t, *J* = 7.7 Hz, 1H), 7.04 (d, *J* = 8.0 Hz, 1H) ppm. ^13^C NMR (151 MHz, DMSO-*d_6_*, 25 °C) *δ* = 156.3, 148.7, 147.8, 142.3, 140.1, 139.6, 136.6, 134.6, 133.7, 129.0, 128.4, 128.3, 128.0, 127.3, 127.2, 126.6, 123.7 (q, *J* = 274.8 Hz), 120.5, 111.8. ^19^F NMR (565 MHz, DMSO-*d_6_*, 25 °C) *δ* = −58.0. MS *m*/*z* 489.3 = [M + H]^+^, calculated for C_21_H_14_F_6_N_2_O_3_S *m*/*z* = 488.0.

*N-(4-(N-([1,1′-Biphenyl]-2-yl)sulfamoyl)phenyl)-3-bromo-1-(3-chloropyridin-2-yl)-1H-pyrazole-5-carboxamide* (**10e**), white solid, m.p. 156–157 °C. ^1^H NMR (600 MHz, DMSO-d*_6_*, 25 °C) *δ* = 10.88 (s, 1H), 9.44 (s, 1H), 8.55 (dd, *J* = 4.7, 1.5 Hz, 1H), 8.27–8.23 (m, 1H), 7.70 (d, *J* = 8.8 Hz, 2H), 7.69–7.66 (m, 1H), 7.53 (d, *J* = 8.8 Hz, 2H), 7.51 (s, 1H), 7.36–7.32 (m, 2H), 7.31 (dt, *J* = 5.5, 2.3 Hz, 1H), 7.29–7.27 (m, 3H), 7.26–7.22 (m, 3H), 7.07–7.03 (m, 1H) ppm. ^13^C NMR (151 MHz, DMSO-*d_6_*, 25 °C) *δ* = 156.3, 148.7, 147.8, 141.9, 140.1, 139.7, 139.2, 139.0, 136.4, 133.7, 131.4, 129.7, 128.5, 128.2, 128.0, 127.5, 127.3, 127.2, 127.1, 120.4, 111.8 ppm. MS *m*/*z* 608.2 = [M + H]^+^, calculated for C_27_H_19_BrClN_5_O_3_S *m*/*z* = 607.0.

*3-Bromo-1-(3-chloropyridin-2-yl)-N-(4-(N-(2-phenoxyphenyl)sulfamoyl)phenyl)-1H-pyrazole-5-carboxamide* (**10f**), white solid, m.p. 177–178 °C. ^1^H NMR (600 MHz, DMSO-d*_6_*, 25 °C) *δ* = 10.82 (s, 1H), 10.82 (s, 1H), 9.84 (s, 1H), 8.54 (dd, *J* = 4.7, 1.4 Hz, 1H), 8.26 (dd, *J* = 8.1, 1.3 Hz, 1H), 7.71–7.60 (m, 5H), 7.48 (s, 1H), 7.36 (dd, *J* = 7.8, 1.7 Hz, 1H), 7.22 (t, *J* = 7.9 Hz, 2H), 7.12–7.03 (m, 3H), 6.67 (dd, *J* = 7.9, 1.4 Hz, 1H), 6.63–6.55 (m, 2H) ppm. ^13^C NMR (151 MHz, DMSO-*d_6_*, 25 °C) *δ* = 156.4, 156.2, 150.2, 148.7, 147.8, 142.1, 140.1, 139.7, 135.8, 130.1, 128.3, 128.1, 127.3, 127.2, 126.5, 123.9, 123.8, 120.4, 119.0, 118.8, 111.7 ppm. MS *m*/*z* 624.2 = [M + H]^+^, calculated for C_27_H_19_BrClN_5_O_4_S *m*/*z* = 623.0.

*3-Bromo-1-(3-chloropyridin-2-yl)-N-(4-(N-(4,5-dimethylisoxazol-3-yl)sulfamoyl)phenyl)-1H-pyrazole-5-carboxmide* (**10g**), white solid, m.p. 171–172 °C. ^1^H NMR (600 MHz, DMSO-d*_6_*, 25 °C) *δ* = 10.91 (s, 1H), 10.66 (s, 1H), 8.53 (dd, *J* = 4.7, 1.5 Hz, 1H), 8.24 (dd, *J* = 8.1, 1.5 Hz, 1H), 7.84–7.79 (m, 4H), 7.66 (dd, *J* = 8.1, 4.7 Hz, 1H), 7.48 (s, 1H), 2.21 (s, 3H), 1.77 (s, 3H) ppm. ^13^C NMR (151 MHz, DMSO-*d_6_*, 25 °C) *δ* = 166.3, 157.1, 156.3, 148.7, 147.8, 142.4, 140.1, 139.7, 135.3, 128.9, 128.2, 127.3, 127.2, 120.4, 111.8, 105.1, 11.2, 6.4 ppm. MS *m*/*z* 651.2 = [M + H]^+^, calculated for C_20_H_16_BrClN_6_O_4_S *m*/*z* = 649.9.

*3-Bromo-1-(3-chloropyridin-2-yl)-N-(4-(N-(2-(trifluoromethyl)phenyl)sulfamoyl)phenyl)-1H-pyrazole-5-carboxamide* (**10h**), white solid, m.p. 181–182 °C. ^1^H NMR (600 MHz, DMSO-d*_6_*, 25 °C) *δ* = 10.91 (s, 1H), 9.92 (s, 1H), 8.55 (dd, *J* = 4.7, 1.5 Hz, 1H), 8.25 (dd, *J* = 8.1, 1.5 Hz, 1H), 7.84 (d, *J* = 8.9 Hz, 2H), 7.76 (d, *J* = 8.8 Hz, 2H), 7.71 (dd, *J* = 7.9, 1.1 Hz, 1H), 7.67 (dd, *J* = 8.1, 4.7 Hz, 1H), 7.60–7.56 (m, 1H), 7.52 (s, 1H), 7.44 (t, *J* = 7.7 Hz, 1H), 7.05 (d, *J* = 8.0 Hz, 1H) ppm. ^13^C NMR (151 MHz, DMSO-*d_6_*, 25 °C) *δ* = 156.3, 148.7, 147.8, 142.3, 140.1, 139.6, 136.6, 134.6, 133.7, 128.9, 128.4, 128.3, 127.9, 127.5, 127.3, 127.2, 126.6, 123.7 (q, *J* = 273.3 Hz), 120.5, 111.8. ^19^F NMR (565 MHz, DMSO-*d_6_*, 25 °C) *δ* = −57.9. MS *m*/*z* 600.5 = [M + H]^+^, calculated for C_22_H_14_F_3_BrClN_5_O_3_S *m*/*z* = 598.9.

*N-(4-(N-([1,1′-Biphenyl]-2-yl)sulfamoyl)phenyl)-2-methyl-[1,1′-biphenyl]-3-carboxamide* (**10i**), white solid, m.p. 197–198 °C. ^1^H NMR (600 MHz, DMSO-d*_6_*, 25 °C) *δ* = 10.79 (s, 1H), 9.40 (s, 1H), 7.83 (d, *J* = 8.7 Hz, 2H), 7.57–7.53 (m, 2H), 7.51–7.46 (m, 3H), 7.43–7.39 (m, 2H), 7.39–7.35 (m, 4H), 7.34–7.33 (m, 2H), 7.30–7.27 (m, 2H), 7.27–7.23 (m, 3H), 7.11–7.08 (m, 1H), 2.25 (s, 3H) ppm. ^13^C NMR (151 MHz, DMSO-*d_6_*, 25 °C) *δ* = 169.1, 143.2, 142.9, 141.3, 139.1, 139.0, 138.7, 135.6, 133.9, 132.4, 131.5, 131.4, 129.7, 129.5, 128.9, 128.5, 128.1, 127.7, 127.5, 127.1, 126.9, 126.7, 126.3, 119.7, 17.7 ppm. MS *m*/*z* 519.4 = [M + H]^+^, calculated for C_32_H_26_N_2_O_3_S *m*/*z* = 518.1.

*2-Methyl-N-(4-(N-(2-phenoxyphenyl)sulfamoyl)phenyl)-[1,1′-biphenyl]-3-carboxamide* (**10j**), white solid, m.p. 170–171 °C. ^1^H NMR (600 MHz, DMSO-d*_6_*, 25 °C) *δ* = 10.76 (s, 1H), 9.84 (s, 1H), 7.80 (d, *J* = 8.7 Hz, 2H), 7.67 (d, *J* = 8.8 Hz, 2H), 7.50–7.46 (m, 3H), 7.42–7.39 (m, 3H), 7.36–7.34 (m, 3H), 7.29–7.27 (m, 2H), 7.10–7.07 (m, 3H), 6.71 (dd, *J* = 7.8, 1.8 Hz, 1H), 6.66 (dd, *J* = 8.6, 0.9 Hz, 2H), 2.24 (s, 3H) ppm. ^13^C NMR (151 MHz, DMSO-*d_6_*, 25 °C) *δ* = 169.1, 156.6, 150.0, 143.4, 143.0, 141.3, 138.6, 135.0, 132.4, 130.1, 129.6, 129.5, 128.8, 128.7, 128.3, 127.7, 127.0, 126.7, 126.3, 126.1, 124.1, 123.9, 119.6, 118.9, 118.8, 17.7 ppm. MS *m*/*z* 535.5 = [M + H]^+^, calculated for C_32_H_26_N_2_O_4_S *m*/*z* = 534.1.

*N-(4-(N-(4,5-Dimethylisoxazol-3-yl)sulfamoyl)phenyl)-2-methyl-[1,1′-biphenyl]-3-carboxamide* (**10k**), white solid, m.p. 156–157 °C. ^1^H NMR (600 MHz, DMSO-d*_6_*, 25 °C) *δ* = 10.84 (s, 1H), 10.65 (s, 1H), 7.94 (d, *J* = 8.8 Hz, 2H), 7.87–7.84 (m, 2H), 7.50–7.47 (m, 3H), 7.43–7.38 (m, 2H), 7.36–7.33 (m, 3H), 2.23 (s, 6H), 1.81 (s, 3H) ppm. ^13^C NMR (151 MHz, DMSO-*d_6_*, 25 °C) *δ* = 169.2, 166.2, 157.2, 143.7, 142.9, 141.3, 138.6, 134.5, 132.5, 131.5, 129.5, 128.9, 128.8, 127.7, 126.7, 126.3, 119.6, 105.0, 17.7, 11.2, 6.4 ppm. MS *m*/*z* 462.4 = [M + H]^+^, calculated for C_25_H_23_N_3_O_4_S *m*/*z* = 461.1.

*2-Methyl-N-(4-(N-(2-(trifluoromethyl)phenyl)sulfamoyl)phenyl)-[1,1′-biphenyl]-3-carboxamide* (**10l**), m.p. 178–180 °C. ^1^H NMR (600 MHz, DMSO-d*_6_*, 25 °C) *δ* = 10.84 (s, 1H), 9.88 (s, 1H), 7.96 (d, *J* = 8.7 Hz, 2H), 7.78 (d, *J* = 8.8 Hz, 2H), 7.72 (d, *J* = 7.2 Hz, 1H), 7.60 (t, *J* = 7.5 Hz, 1H), 7.50–7.44 (m, 4H), 7.40 (dd, *J* = 14.0, 7.4 Hz, 2H), 7.36–7.33 (m, 3H), 7.08 (d, *J* = 8.1 Hz, 1H), 2.24 (s, 3H) ppm. ^13^C NMR (151 MHz, DMSO-*d_6_*, 25 °C) *δ* = 169.2, 143.5, 143.0, 141.3, 138.6, 135.8, 134.8, 133.8, 132.5, 131.5, 129.5, 128.9, 128.8, 128.4, 127.8, 127.7, 127.5, 126.7, 126.5, 126.3, 123.7 (q, *J* = 273.3 Hz), 119.8, 17.7. ^19^F NMR (565 MHz, DMSO-*d_6_*, 25 °C) *δ* = −57.9. MS *m*/*z* 511.4 = [M + H]^+^, calculated for C_27_H_21_F_3_N_2_O_3_S *m*/*z* = 510.1.

*N-(4-(N-([1,1′-Biphenyl]-2-yl)sulfamoyl)phenyl)-4′-(trifluoromethyl)-[1,1′-biphenyl]-2-carboxamide* (**10m**), white solid, m.p. 163–164 °C. ^1^H NMR (600 MHz, DMSO-d*_6_*, 25 °C) *δ* = 10.76 (s, 1H), 9.40 (s, 1H), 7.80 (d, *J* = 8.2 Hz, 2H), 7.73 (dd, *J* = 7.5, 1.1 Hz, 1H), 7.71–7.67 (m, 3H), 7.65–7.61(m, 3H), 7.58 (d, *J* = 7.6 Hz, 1H), 7.48 (d, *J* = 8.8 Hz, 2H), 7.35–7.28 (m, 5H), 7.27–7.24 (m, 1H), 7.21–7.17 (m, 2H), 7.14 (d, *J* = 9.3 Hz, 1H) ppm. ^13^C NMR (151 MHz, DMSO-*d_6_*, 25 °C) *δ* = 168.3, 144.6, 142.9, 139.1, 139.0, 138.6, 137.0, 135.6, 133.8, 131.3, 130.9, 130.6, 129.7, 129.6, 128.6, 128.5, 128.2, 127.9, 127.4, 127.2, 127.1, 126.7, 126.6, 124.7 (q, *J* = 272.0 Hz), 119.7. MS *m*/*z* 573.6 = [M + H]^+^, calculated for C_32_H_23_F_3_N_2_O_3_S *m*/*z* = 572.1.

*N-(4-(N-(2-Phenoxyphenyl)sulfamoyl)phenyl)-4′-(trifluoromethyl)-[1,1′-biphenyl]-2-carboxamide* (**10n**), white solid, m.p. 173–174 °C. ^1^H NMR (600 MHz, DMSO-d*_6_*, 25 °C) *δ* = 10.74 (s, 1H), 9.82 (s, 1H), 7.73 (d, *J* = 8.2 Hz, 2H), 7.69–7.67 (m, 1H), 7.66–7.63 (m, 2H), 7.63–7.56 (m, 6H), 7.54 (d, *J* = 7.6 Hz, 1H), 7.37 (dd, *J* = 7.8, 1.9 Hz, 1H), 7.25–7.21 (m, 2H), 7.11–7.03 (m, 3H), 6.70 (dd, *J* = 7.9, 1.6 Hz, 1H), 6.64 (dd, *J* = 8.6, 0.9 Hz, 2H) ppm. ^13^C NMR (151 MHz, DMSO-*d_6_*, 25 °C) *δ* = 168.3, 156.6, 149.9, 144.7, 143.1, 138.6, 136.9, 135.1, 130.9, 130.7, 130.1, 129.6, 128.6, 128.4, 128.2, 126.9, 125.7 (q, *J* = 3.0 Hz), 124.1 (q, *J* = 272.0 Hz), 123.8, 119.6, 118.9, 118.8. ^19^F NMR (565 MHz, DMSO-*d_6_*, 25 °C) *δ* = −60.9. MS *m*/*z* 589.6 = [M + H]^+^, calculated for C_32_H_23_F_3_N_2_O_4_S *m*/*z* = 588.1.

*N-(4-(N-(4,5-Dimethylisoxazol-3-yl)sulfamoyl)phenyl)-4′-(trifluoromethyl)-[1,1′-biphenyl]-2-carboxamide* (**10o**), white solid, m.p. 199–200 °C. ^1^H NMR (600 MHz, DMSO-d*_6_*, 25 °C) *δ* = 10.82 (s, 1H), 10.61 (s, 1H), 7.78–7.76 (m, 3H), 7.75–7.73 (m, 3H), 7.69 (dd, *J* = 7.6, 1.1 Hz, 1H), 7.64–7.62 (m, 2H), 7.58 (td, *J* = 7.5, 1.2 Hz, 1H), 7.55–7.53 (m, 2H), 2.22 (s, 3H) ppm. ^13^C NMR (151 MHz, DMSO-*d_6_*, 25 °C) *δ* = 169.4, 168.3, 166.2, 157.2, 144.6, 143.5, 140.5, 138.6, 136.8, 134.5, 132.2, 131.8, 130.9, 128.8, 128.3 (q, *J* = 31.7 Hz), 125.8 (q, *J* = 3.0 Hz), 124.7 (q, *J* = 273.0 Hz), 119.6, 105.0, 11.2, 6.3. ^19^F NMR (565 MHz, DMSO-*d_6_*, 25 °C) *δ* = −60.9. MS *m*/*z* 516.3 = [M + H]^+^, calculated for C_25_H_20_F_3_N_3_O_4_S *m*/*z* = 515.1.

*4′-(Trifluoromethyl)-N-(4-(N-(2-(trifluoromethyl)phenyl)sulfamoyl)phenyl)-[1,1′-biphenyl]-2-carboxamide* (**10p**), white solid, m.p. 160–161 °C. ^1^H NMR (600 MHz, DMSO-d*_6_*, 25 °C) *δ* = 10.77 (s, 1H), 9.86 (s, 1H), 7.76–7.73 (m, 4H), 7.72–7.69 (m, 4H), 7.64–7.62 (m, 2H), 7.61–7.57 (m, 2H), 7.56–7.53 (m, 2H), 7.46–7.44 (m, 1H), 7.06 (d, *J* = 8.0 Hz, 1H) ppm. ^13^C NMR (151 MHz, DMSO-*d_6_*, 25 °C) *δ* = 168.4, 144.6, 143.2, 138.6, 136.9, 135.8, 134.7, 133.7, 131.8, 130.9, 130.7, 130.1, 129.7, 129.6, 128.8, 127.8, 127.5, 126.6 (q, *J* = 28.7 Hz), 125.7 (q, *J* = 3.8 Hz), 125.4 (q, *J* = 4.5 Hz), 123.8, 123.7 (q, *J* = 273.3 Hz), 122.9 (q, *J* = 274.0 Hz), 119.7. ^19^F NMR (565 MHz, DMSO-*d_6_*, 25 °C) *δ* = −58.0, −60.9. MS *m*/*z* 565.5 = [M + H]^+^, calculated for C_27_H_18_F_6_N_2_O_3_S *m*/*z* = 564.0.

*2-(6-Chloro-9H-carbazol-2-yl)-N-(4-(N-(2-(trifluoromethyl)phenyl)sulfamoyl)phenyl)propanamide* (**10q**), white solid, m.p. 159–160 °C. ^1^H NMR (600 MHz, DMSO-d*_6_*, 25 °C) *δ* = 11.39 (s, 1H), 10.52 (s, 1H), 9.83 (s, 1H), 8.17 (d, *J* = 2.1 Hz, 1H), 8.11 (d, *J* = 8.1 Hz, 1H), 7.81 (d, *J* = 8.9 Hz, 2H), 7.72–7.65 (m, 3H), 7.56–7.51 (m, 2H), 7.48 (d, *J* = 8.6 Hz, 1H), 7.43–7.34 (m, 2H), 7.23 (dd, *J* = 8.2, 1.4 Hz, 1H), 7.00 (d, *J* = 8.0 Hz, 1H), 4.05–4.02 (m, 1H), 1.52 (d, *J* = 7.0 Hz, 3H) ppm. ^13^C NMR (151 MHz, DMSO-*d_6_*, 25 °C) *δ* = 173.6, 141.0, 140.4, 138.8, 133.6, 128.4, 127.5 (q, *J* = 4.5 Hz), 125.6, 124.1, 123.3, 121.1, 121.0, 120.1, 119.3, 119.2, 112.8, 110.1, 47.0, 19.5. ^19^F NMR (565 MHz, DMSO-*d_6_*, 25 °C) *δ* = −57.9. MS *m*/*z* 572.4 = [M + H]^+^, calculated for C_28_H_21_ClF_3_N_3_O_3_S *m*/*z* = 571.0.

*2-(6-Chloro-9H-carbazol-2-yl)-N-(4-(N-(2-phenoxyphenyl)sulfamoyl)phenyl)propanamide* (**10r**), white solid, m.p. 166–167 °C. ^1^H NMR (600 MHz, DMSO-d*_6_*, 25 °C) *δ* = 10.87 (s, 1H), 9.45 (s, 1H), 8.56 (d, *J* = 2.9 Hz, 1H), 8.26 (d, *J* = 7.7 Hz, 1H), 7.77–7.66 (m, 3H), 7.59–7.50 (m, 3H), 7.35–7.31 (m, 2H), 7.28–7.22 (m, 4H), 7.06 (s, 1H), 4.04 (q, *J* = 7.0 Hz, 1H), 1.21 (d, *J* = 7.0 Hz, 3H) ppm. ^13^C NMR (151 MHz, DMSO-*d_6_*, 25 °C) *δ* = 170.8, 156.3, 148.8, 147.8, 142.0, 140.1, 139.8, 139.2, 139.1, 136.4, 133.7, 131.4, 129.7, 128.5, 128.2, 128.1, 127.5, 127.4, 127.3, 127.2, 120.4, 111.8, 60.2, 21.2 ppm. MS *m*/*z* 596.4 = [M + H]^+^, calculated for C_33_H_26_ClN_3_O_4_S *m*/*z* = 595.1.

Bioassays: The in vitro cytotoxicity of the novel carbazole derivatives against human melanoma (A875), human hepatocellular liver carcinoma (HepG2), and a subclone of African green monkey kidney cell line MA-104 (MARC145) cell lines was evaluated using the MTT assay. All data were analyzed with SPSS software, and the 50% inhibitory concentrations (IC_50_) of each compound for the different cell lines were determined. All assays were performed in triplicate on three independent experiments.

## Figures and Tables

**Table 1:** In vitro cytotoxic activities of target compounds against tested cell lines.

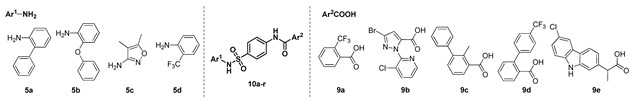						
**Entry**	**Compd.** **No.**	**Substrates**		**In Vitro cytotoxicity IC_50_ *^a^*** **(μg/mL)**		
		**Ar^1^NH_2_**	**Ar^2^CO_2_H**	**A875 *^b^***	**HepG2 *^b^***	**MARC145 *^b^***
1	**10a**	5a	9a	>40	22.06 ± 3.99	13.16 ± 2.49
2	**10b**	5b	9a	>40	>40	>40
3	**10c**	5c	9a	>40	>40	>40
4	**10d**	5d	9a	>40	33.08 ± 2.13	24.89 ± 4.45
5	**10e**	5a	9b	>40	>40	>40
6	**10f**	5b	9b	>40	>40	11.79 ± 2.93
7	**10g**	5c	9b	>40	>40	>40
8	**10h**	5d	9b	16.79 ± 1.14	14.36 ± 1.89	6.75 ± 0.89
9	**10i**	5a	9c	>40	>40	>40
10	**10j**	5b	9c	>40	>40	>40
11	**10k**	5c	9c	>40	34.69 ± 2.94	>40
12	**10l**	5d	9c	10.76 ± 2.28	9.03 ± 1.49	5.62 ± 0.98
13	**10m**	5a	9d	>40	>40	>40
14	**10n**	5b	9d	>40	>40	22.56 ± 2.78
15	**10o**	5c	9d	>40	>40	>40
16	**10p**	5d	9d	20.42 ± 3.99	9.60 ± 0.88	8.50 ± 2.55
17	**10q**	5d	9e	4.19 ± 0.78	3.55 ± 0.63	2.95 ± 0.78
18	**10r**	5b	9e	15.15 ± 2.40	8.84 ± 1.68	5.96 ± 1.33
19	**NC1**	-	-	27.17 ± 5.39	-	25.80 ± 10.40
20	**NC2**	-	-	>40	-	>40
21	**5-FU** *^c^*	**-**	**-**	11.76 ± 2.03	15.14 ± 1.06	13.05 ± 1.32

*^a^* IC_50_—Compound concentration required to inhibit tumor cell proliferation by 50%. ^*b*^ Abbreviations: A875—human melanoma cell line; HepG2—human hepatocellular liver carcinoma cell line; MARC145—a subclone of African green monkey kidney cell line MA-104. ^*c*^ Used as a positive control.

## Data Availability

The data presented in this research are available on request from the corresponding authors.

## Supplementary Materials

The following are available online. Instrumentation and chemicals, and Spectroscopy for target compounds.

## Sample Availability

Samples of the compounds **8a**–**d** are available from the authors.
