# Supplementary material for: Design, Synthesis, and Cytotoxic Activity of Novel Natural Arylsulfonamide-Inspired Molecules
Source: Molecules. 2022 Feb 22;27(5):1479. doi: 10.3390/molecules27051479 (PMC8911723; doi:10.3390/molecules27051479)
Supplement: Supplementary file 1 [file molecules-27-01479-s001.zip › molecules-1576278-supplementary.pdf]

Design, Synthesis, and Cytotoxic Activity of Novel Natural Arylsulfonamides-Inspired Molecules

Supplementary Materials

Experimental section

Instrumentation and chemicals

All melting points (m.p.) were measured using a digital model X-5 apparatus (Shanghai Instrument Physical Optics Instrument Co., LTD, Shanghai, China), and were uncorrected. ^{1}H NMR, ^{13}C NMR, and ^{19}F NMR spectra were recorded on a Bruker Avance III 600 MHz FT-NMR spectrometer (Bruker, Billerica, MA, USA), using $CDCl_3$ or DMSO-d_6 as the solvent and tetramethylsilane (TMS) as the internal standard. Chemical shifts are reported in δ (parts per million) values, and coupling constants ^nJ are reported in Hz. Mass spectra were recorded on a Waters ACQUITY UPLC® H-Class PDA (Waters®) instrument (Waters®, Milford, MA, USA). Thin-layer chromatography (TLC) was carried out on precoated GF254 silica gel plates (Qingdao Haiyang Chemical, Qingdao, China), and spots were visualized with ultraviolet light. All commercially available starting materials and reagents were used without further purification, unless otherwise specified.

Spectroscopy for target compounds

Structures of natural compounds **NC1**, **NC2**, and target compounds **10a-r** were confirmed by their ^{1}H NMR, ^{13}C NMR, ESI-MS and their ^{1}H NMR, ^{13}C NMR and ESI-MS were consistent with the assigned structures. The typical ^{1}H NMR and ^{13}C NMR for synthesized compounds have been presented in the following, which can confirm the result.

Compd NC1

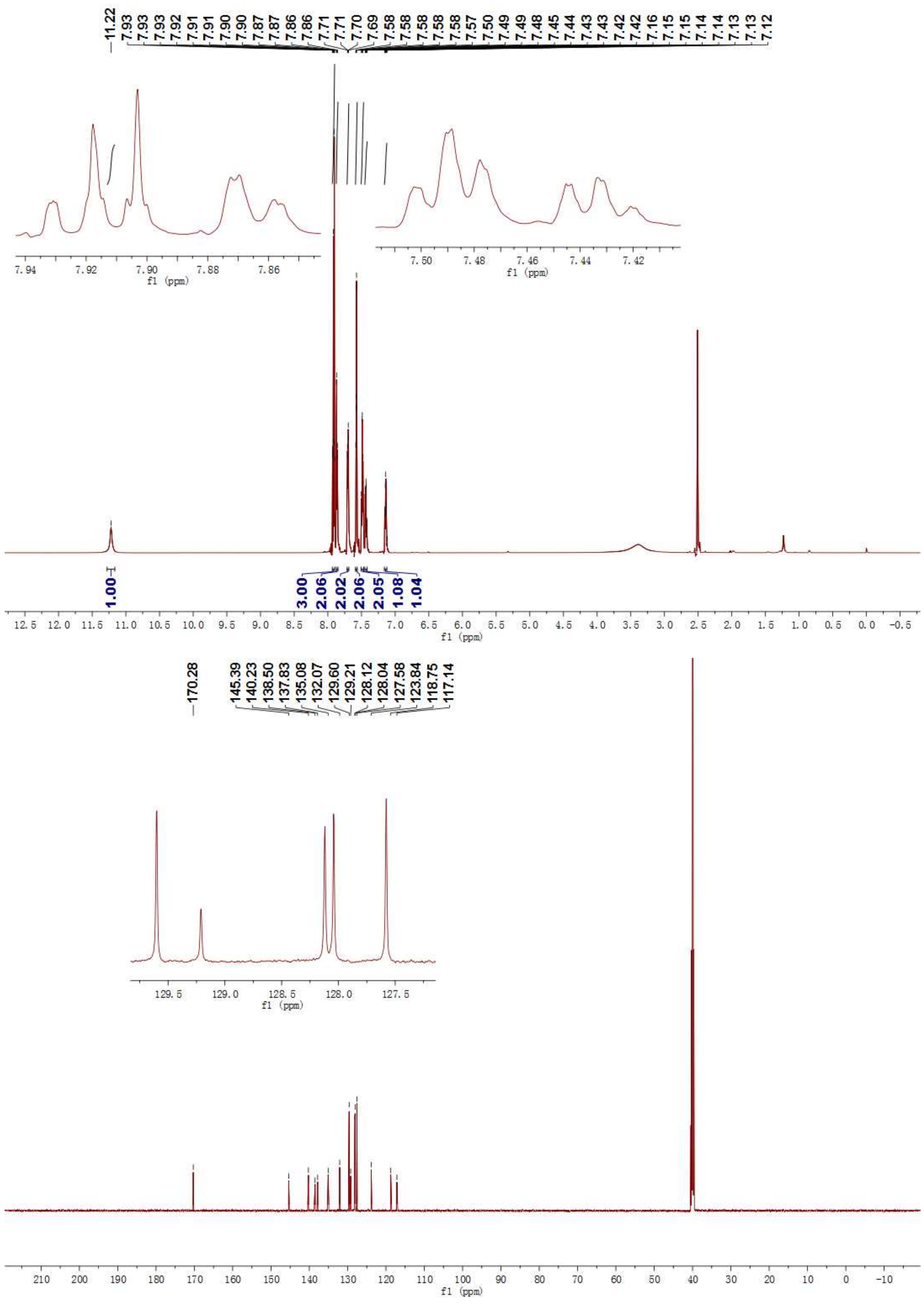
11.22
7.93
7.93
7.93
7.92
7.91
7.91
7.90
7.90
7.87
7.86
7.86
7.71
7.71
7.70
7.69
7.58
7.58
7.58
7.58
7.58
7.57
7.50
7.49
7.49
7.48
7.45
7.44
7.43
7.43
7.42
7.42
7.16
7.15
7.15
7.14
7.14
7.13
7.12
1.00
3.00
2.06
2.02
2.06
2.05
1.08
1.04
f1 (ppm)
170.28
145.39
140.23
138.50
137.83
135.08
132.07
129.60
129.21
128.12
128.04
127.58
123.84
118.75
117.14
f1 (ppm)

Compd NC2

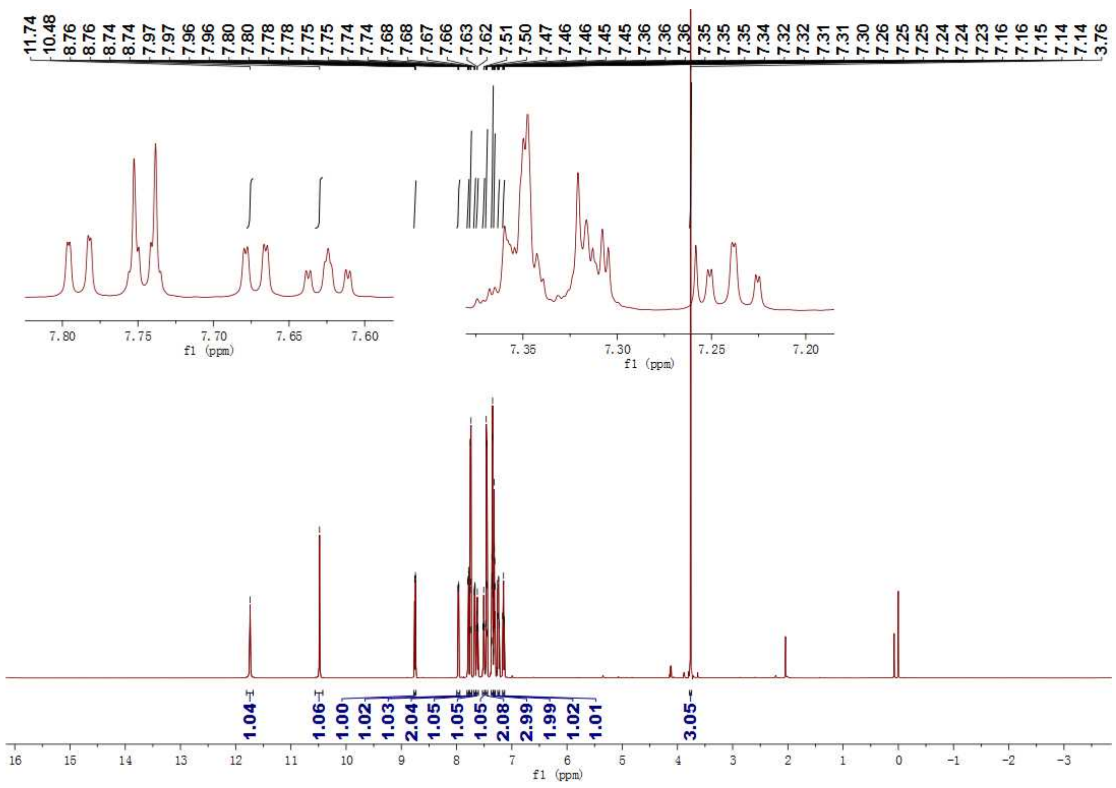
11.74
10.48
8.76
8.76
8.74
8.74
7.97
7.97
7.96
7.96
7.80
7.80
7.78
7.78
7.75
7.75
7.74
7.74
7.68
7.68
7.67
7.66
7.63
7.62
7.51
7.50
7.47
7.46
7.46
7.45
7.45
7.36
7.36
7.36
7.35
7.35
7.35
7.34
7.32
7.32
7.31
7.31
7.30
7.26
7.25
7.25
7.24
7.24
7.23
7.16
7.15
7.14
7.14
3.76
1.04
1.06
1.00
1.02
1.03
2.04
1.05
1.05
1.05
2.08
2.99
1.99
1.02
1.01
3.05
f1 (ppm)

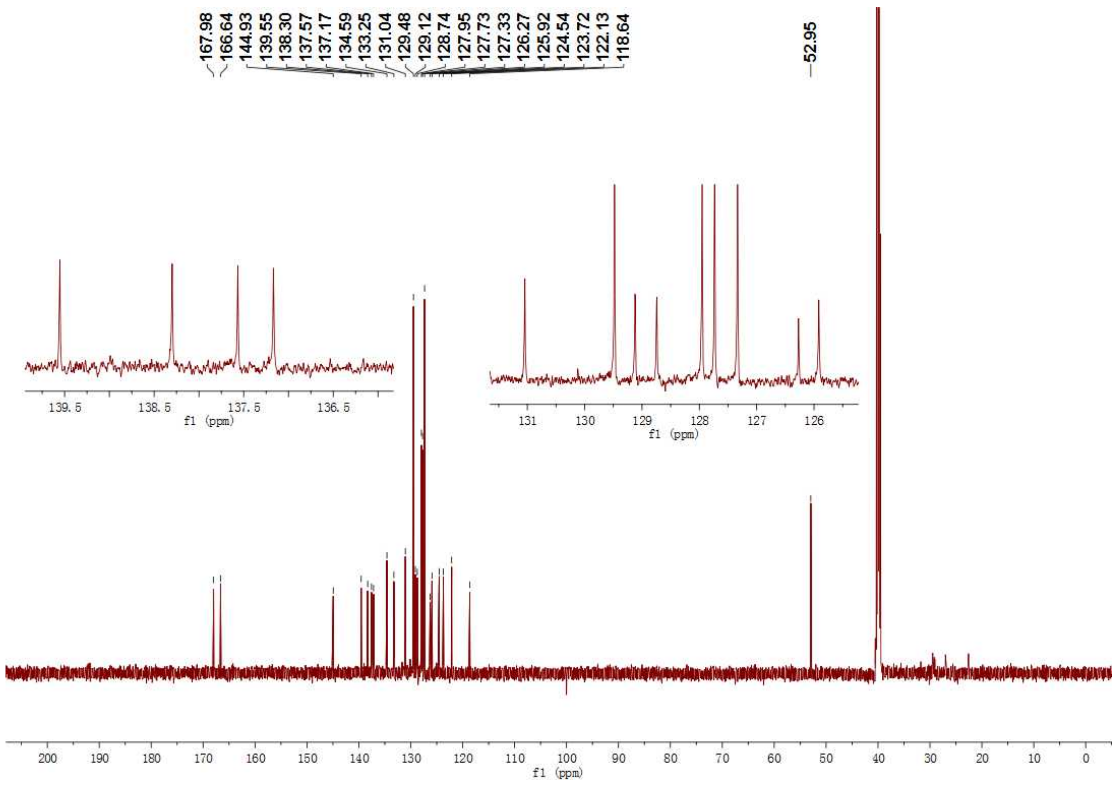
167.98
166.64
144.93
139.55
138.30
137.57
137.17
134.59
133.25
131.04
129.48
129.12
128.74
127.95
127.73
127.33
126.27
125.92
124.54
123.72
122.13
118.64
52.95
f1 (ppm)

Compd 10a

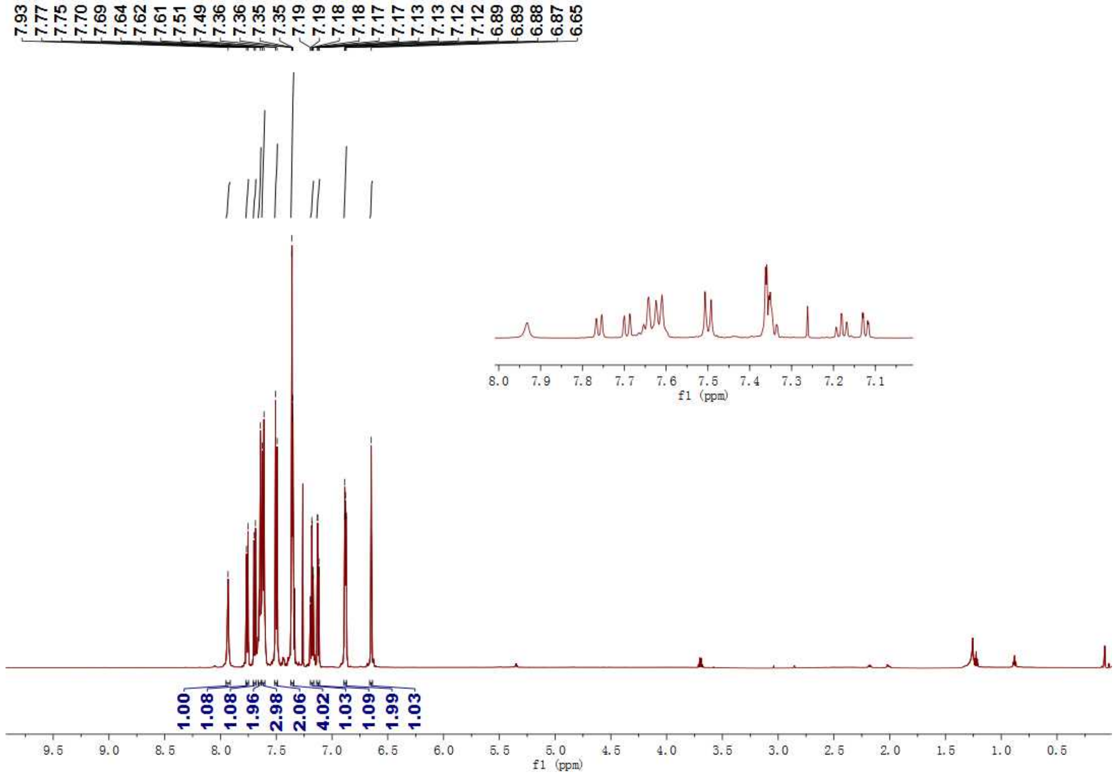

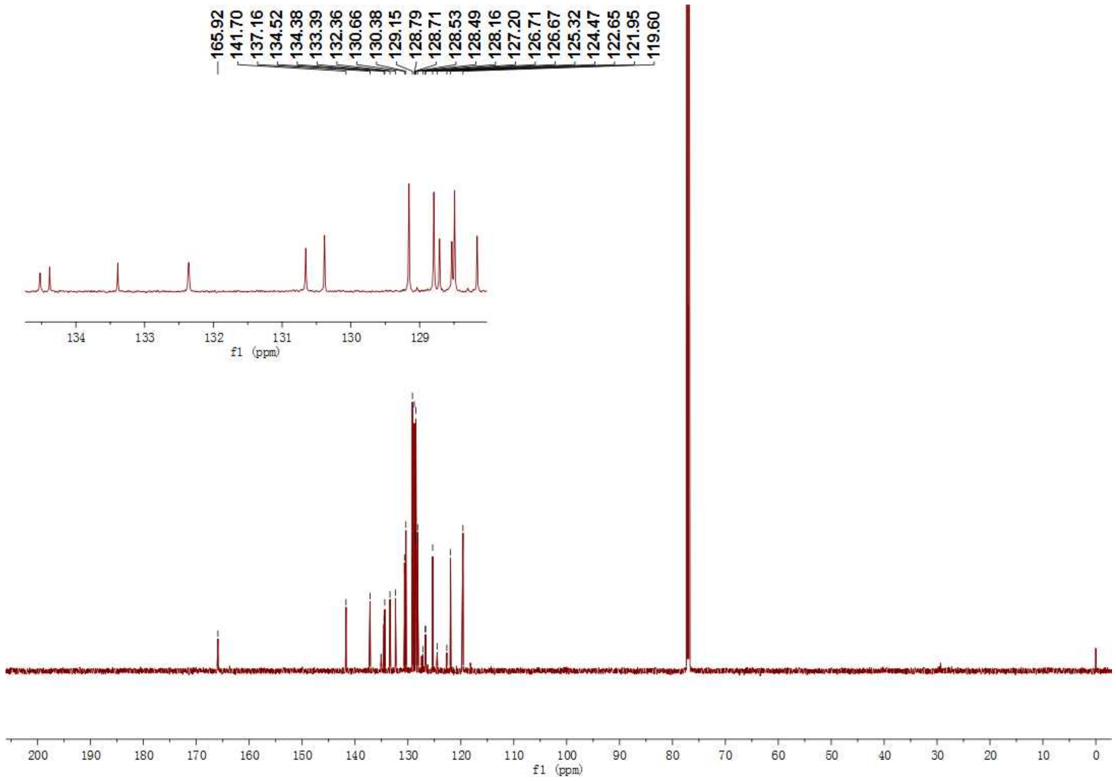

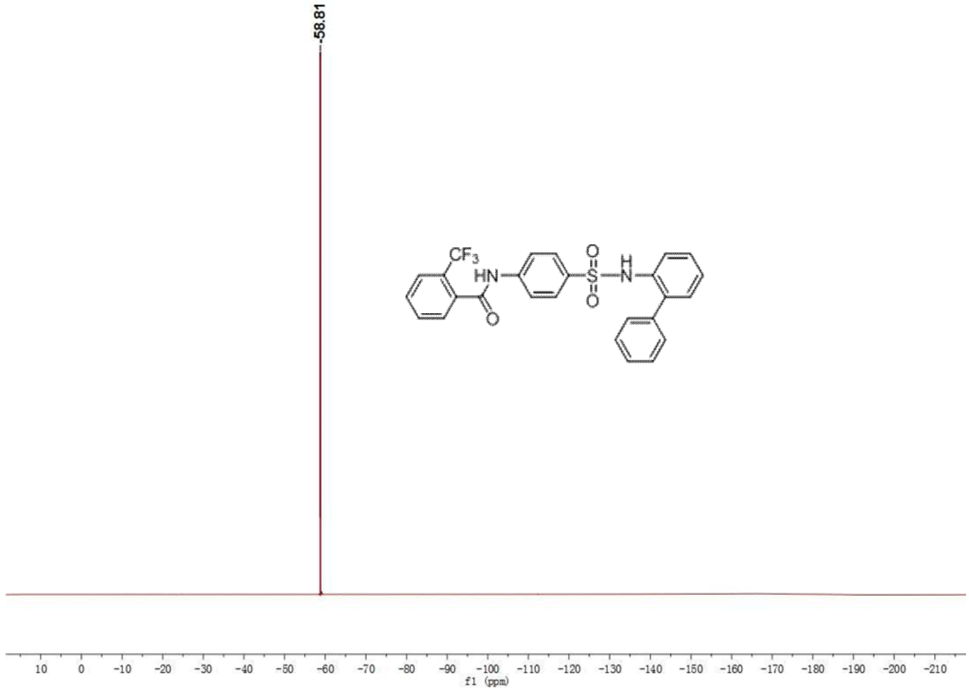
-58.81
CF3
HN
O
S
H
N
f1 (ppm)

Compd 10b

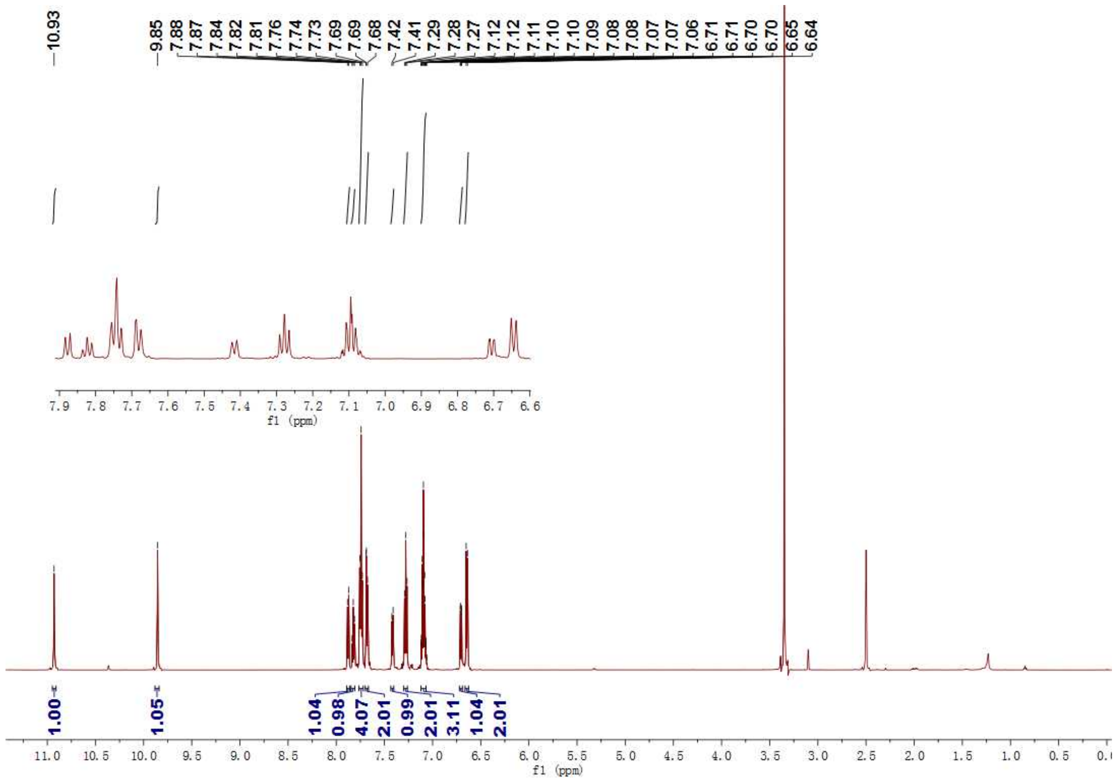
10.93
9.85
7.88
7.87
7.84
7.82
7.81
7.76
7.74
7.73
7.69
7.68
7.42
7.41
7.29
7.28
7.27
7.12
7.12
7.11
7.10
7.09
7.08
7.07
7.07
7.06
6.71
6.71
6.70
6.65
6.64
1.00
1.05
1.04
0.98
4.07
2.01
0.99
2.01
3.11
1.04
2.01
f1 (ppm)

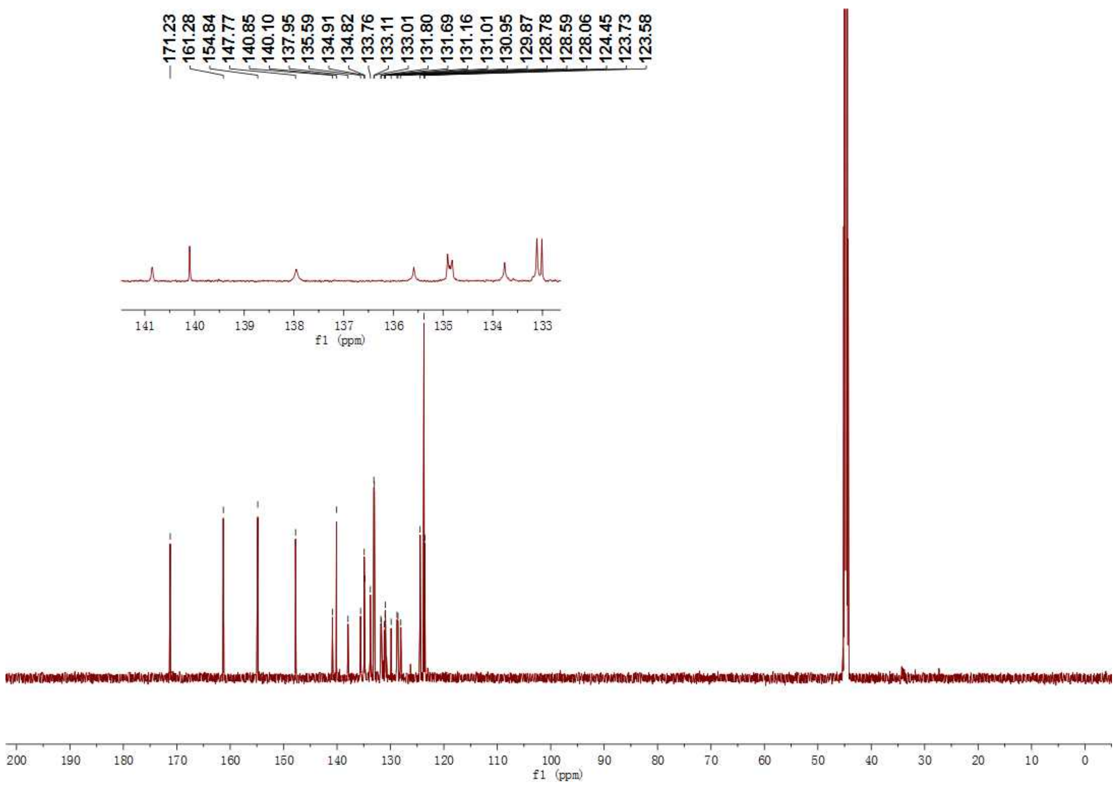
171.23
161.28
154.84
147.77
140.85
140.10
137.95
135.59
134.91
134.82
133.76
133.11
133.01
131.80
131.69
131.16
131.01
130.95
129.87
128.78
128.59
128.06
124.45
123.73
123.58
f1 (ppm)

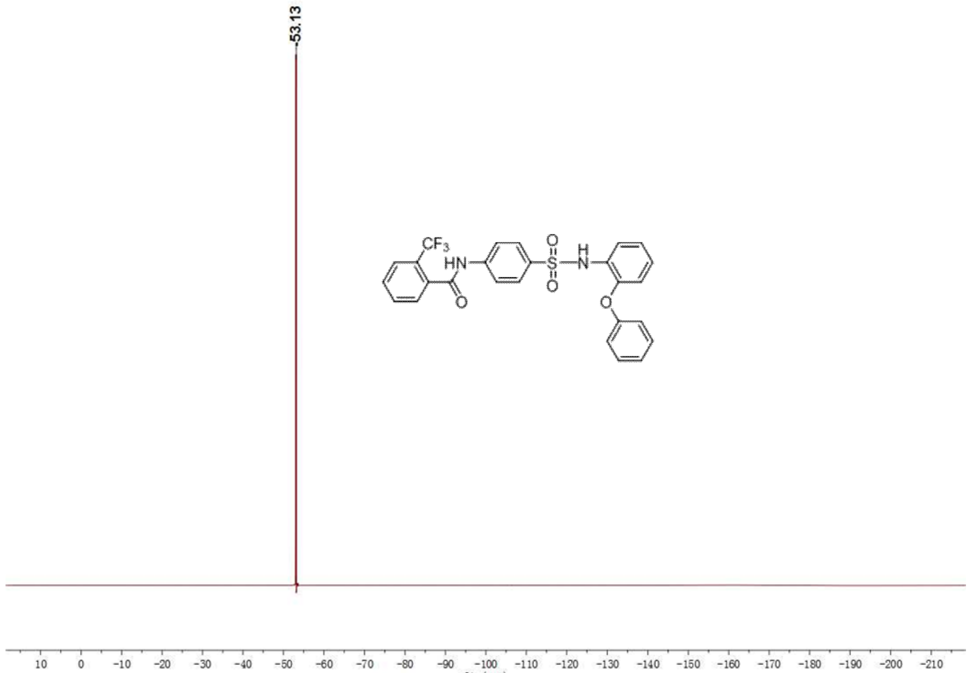
-53.13
CF_3
HN
O
S
H
N
f1 (ppm)

Compd 10c

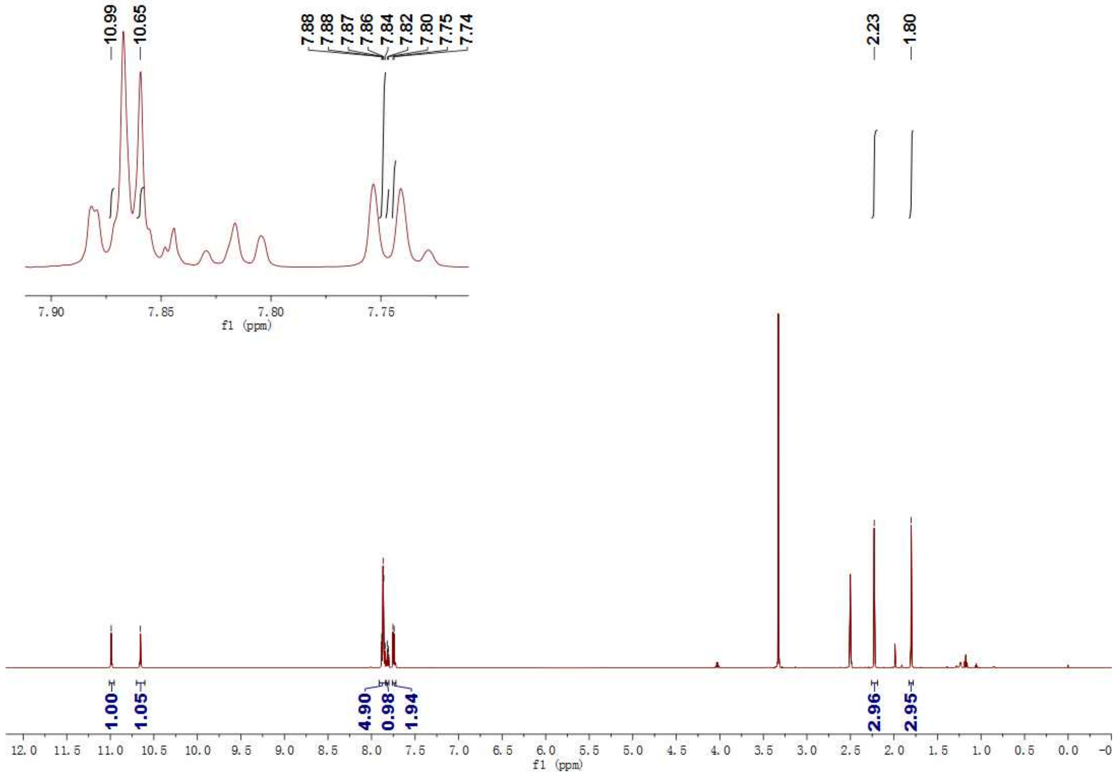

10.99
10.65
7.88
7.88
7.87
7.86
7.84
7.82
7.80
7.75
7.74
2.23
1.80
1.00
1.05
4.90
0.98
1.94
2.96
2.95
f1 (ppm)

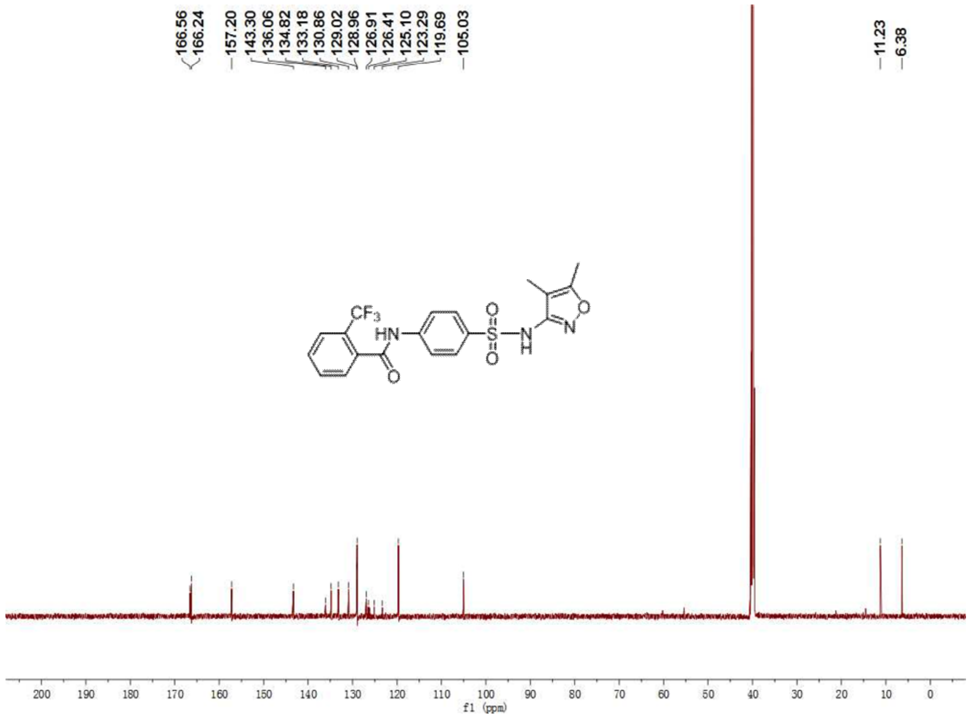

166.56
166.24
157.20
143.30
136.06
134.82
133.18
130.86
129.02
128.96
126.91
126.41
125.10
123.29
119.69
105.03
11.23
6.38
CF3
HN
O
S
N
H
f1 (ppm)

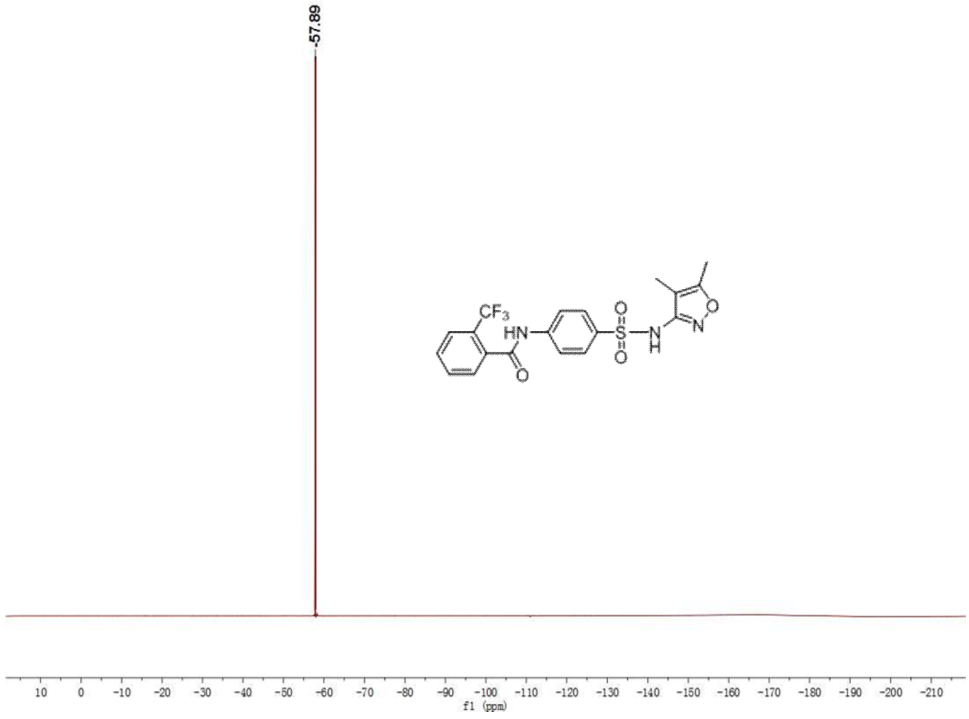
-57.89
CF3
HN
O
S
N
H
f1 (ppm)

Compd 10d

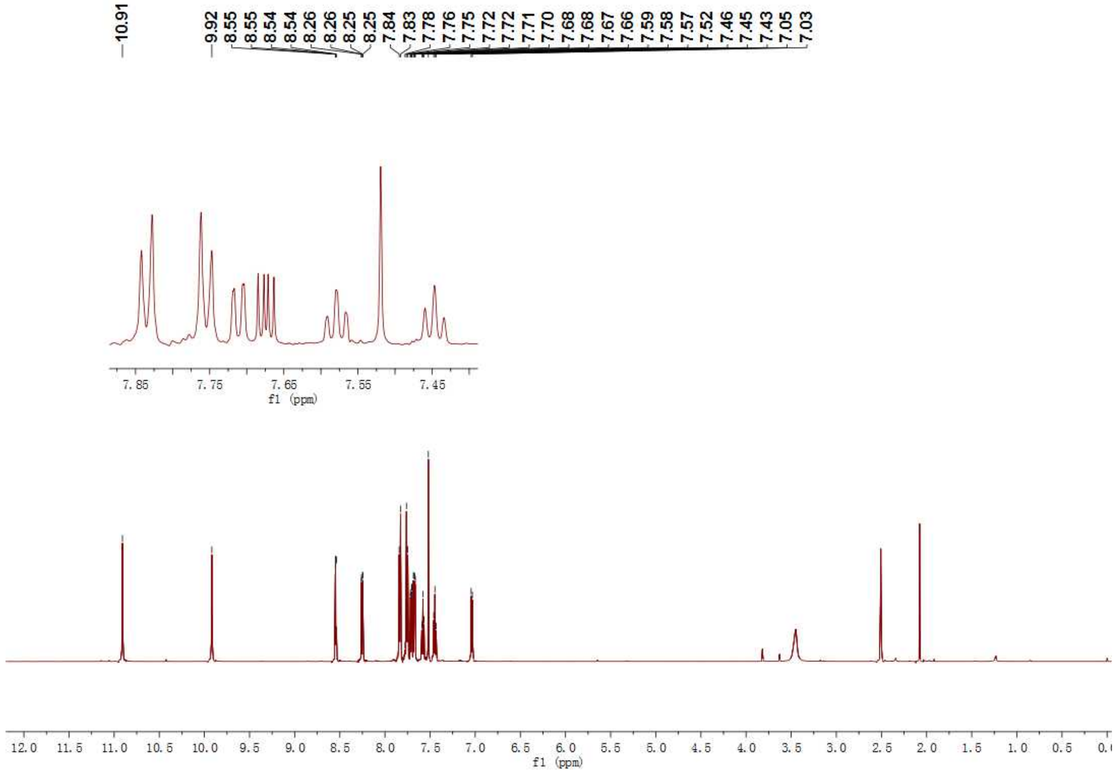
10.91
9.92 8.55 8.55 8.54 8.54 8.26 8.26 8.25 8.25 7.84 7.83 7.78 7.76 7.75 7.72 7.72 7.71 7.70 7.68 7.68 7.67 7.66 7.59 7.58 7.57 7.52 7.46 7.45 7.43 7.05 7.03
f1 (ppm)

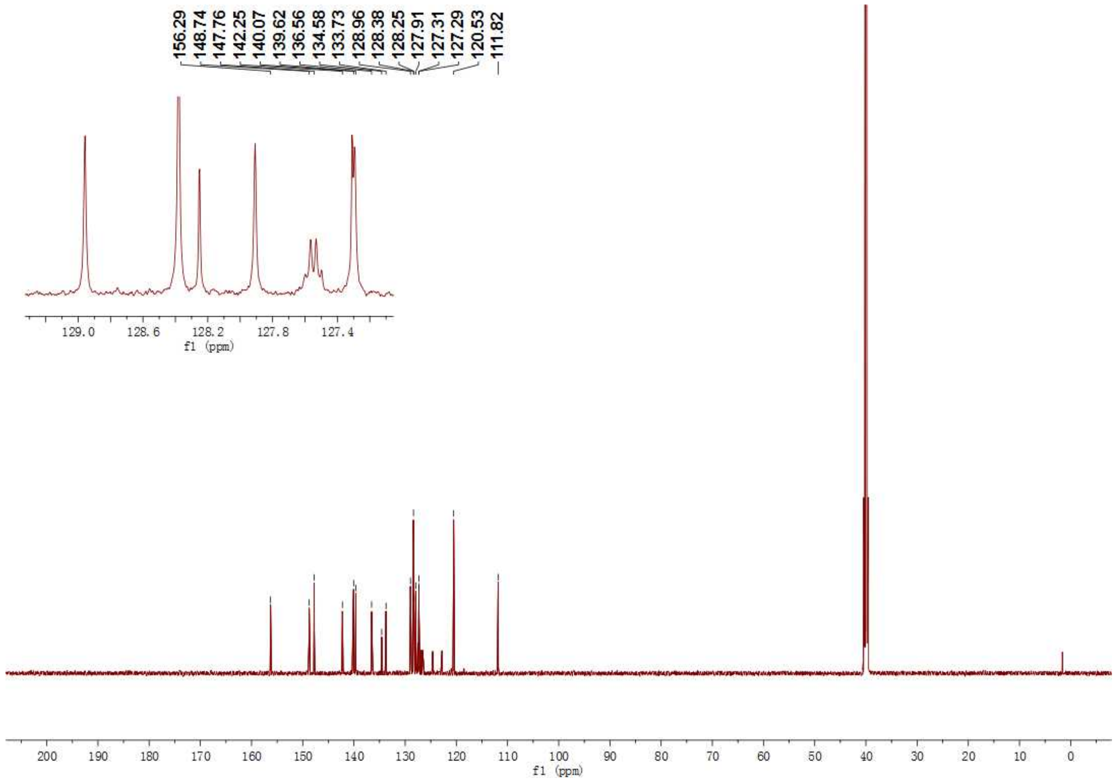
156.29
148.74
147.76
142.25
140.07
139.62
136.56
134.58
133.73
128.96
128.38
128.25
127.91
127.31
127.29
120.53
111.82
f1 (ppm)

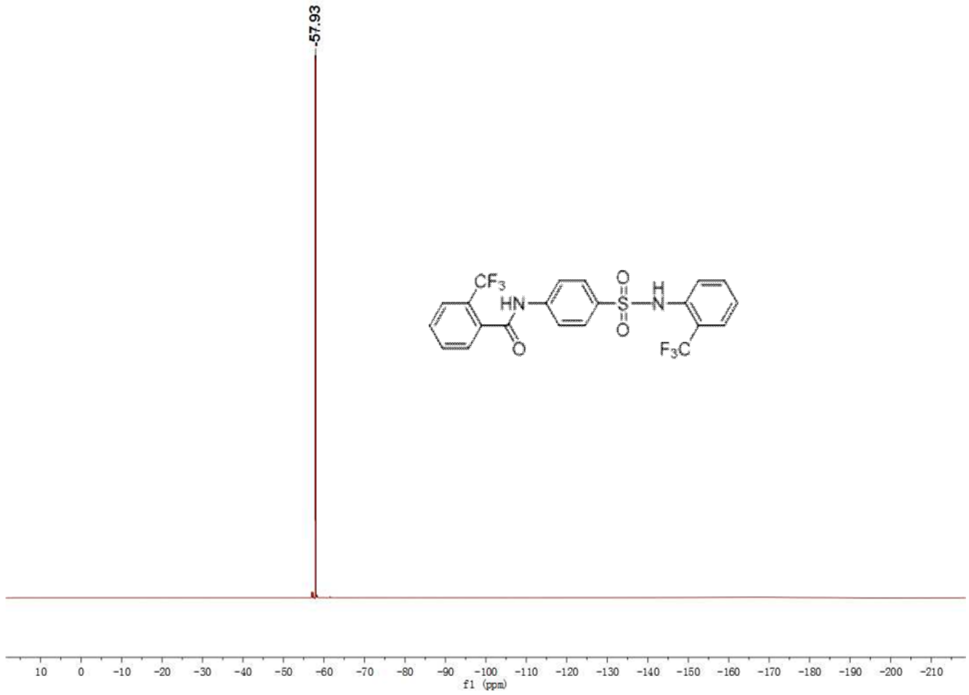
-57.93
CF3
HN
O
S
H
N
F3C
f1 (ppm)

Compd 10e

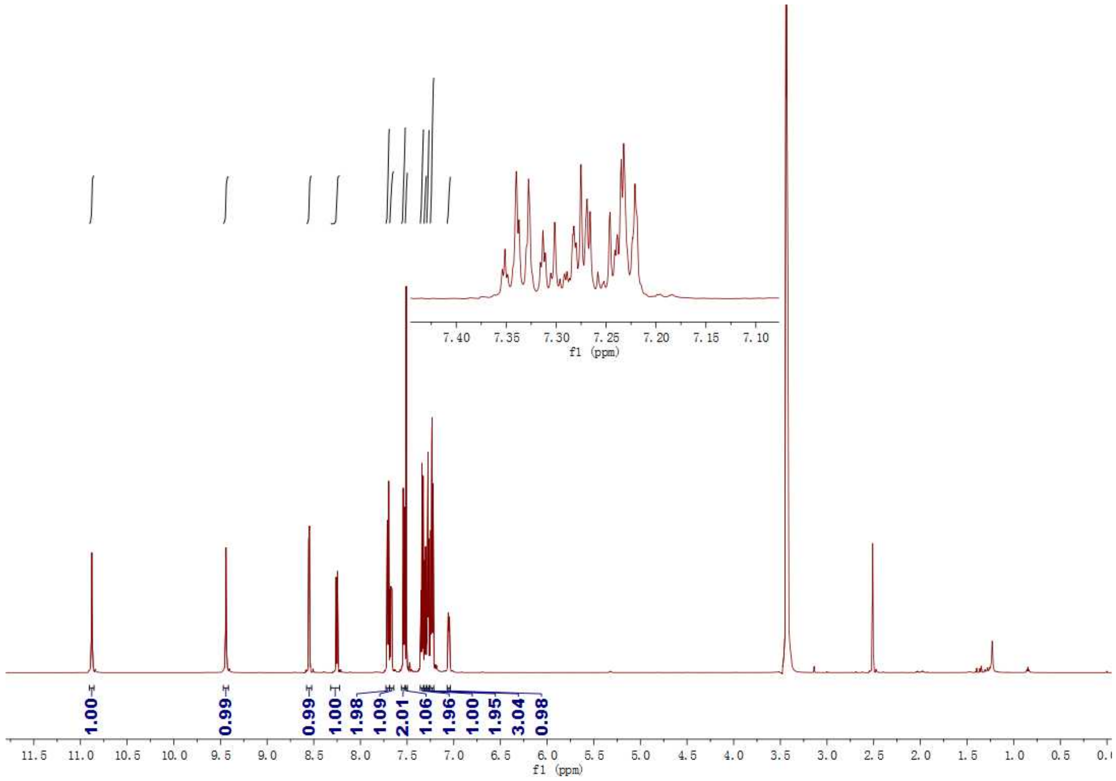
1.00
0.99
0.99
1.00
1.98
1.09
2.01
1.06
1.96
1.00
1.95
3.04
0.98
f1 (ppm)

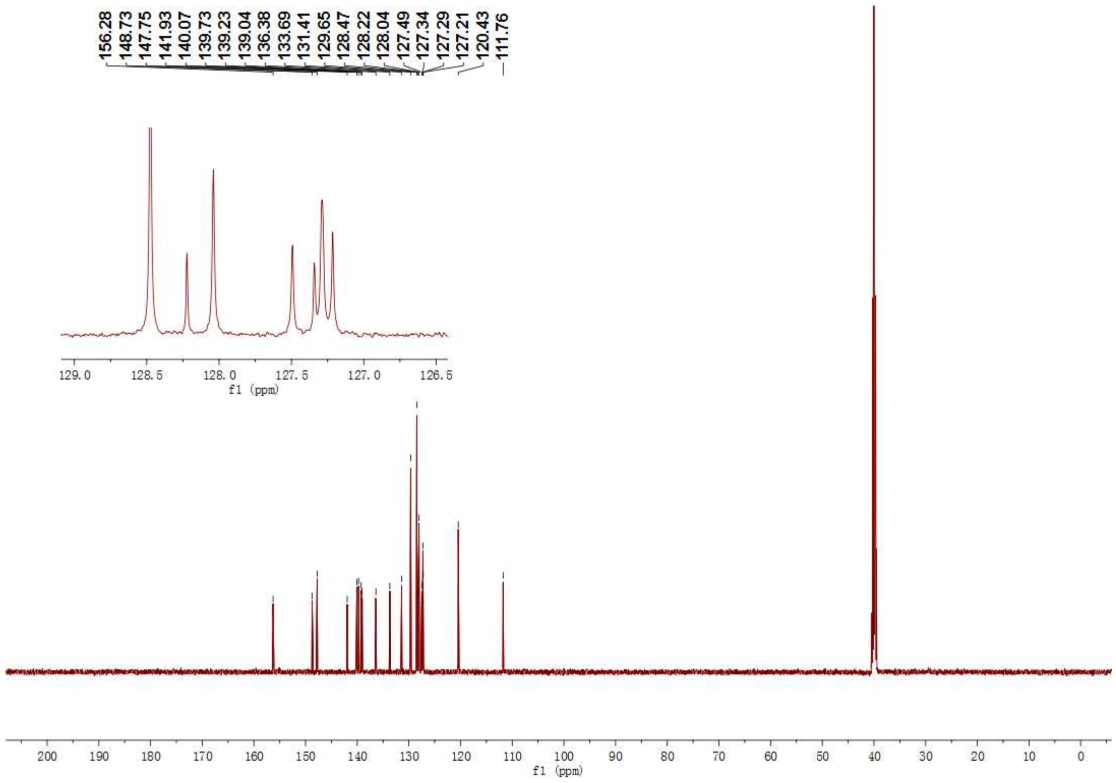
156.28
148.73
147.75
141.93
140.07
139.73
139.23
139.04
136.38
133.69
131.41
129.65
128.47
128.22
128.04
127.49
127.34
127.29
127.21
120.43
111.76
f1 (ppm)

Compd 10f

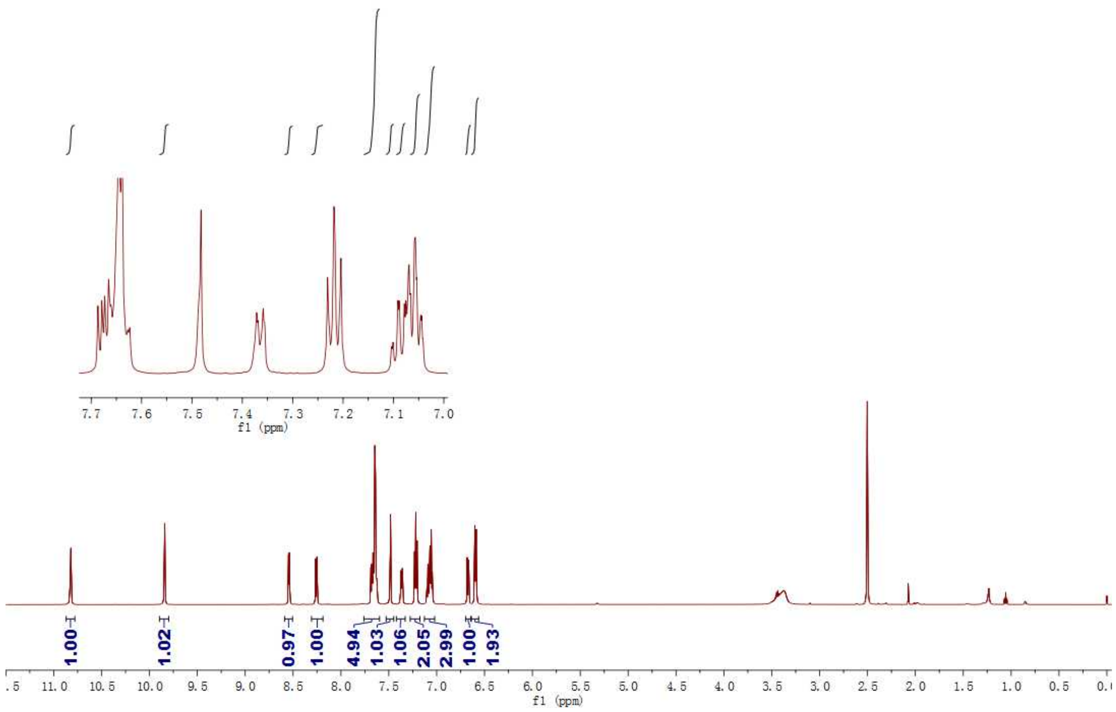

7.7 7.6 7.5 7.4 7.3 7.2 7.1 7.0
f1 (ppm)
1.00
1.02
0.97
1.00
4.94
1.03
1.06
2.05
2.99
1.00
1.93
11.0 10.5 10.0 9.5 9.0 8.5 8.0 7.5 7.0 6.5 6.0 5.5 5.0 4.5 4.0 3.5 3.0 2.5 2.0 1.5 1.0 0.5
f1 (ppm)

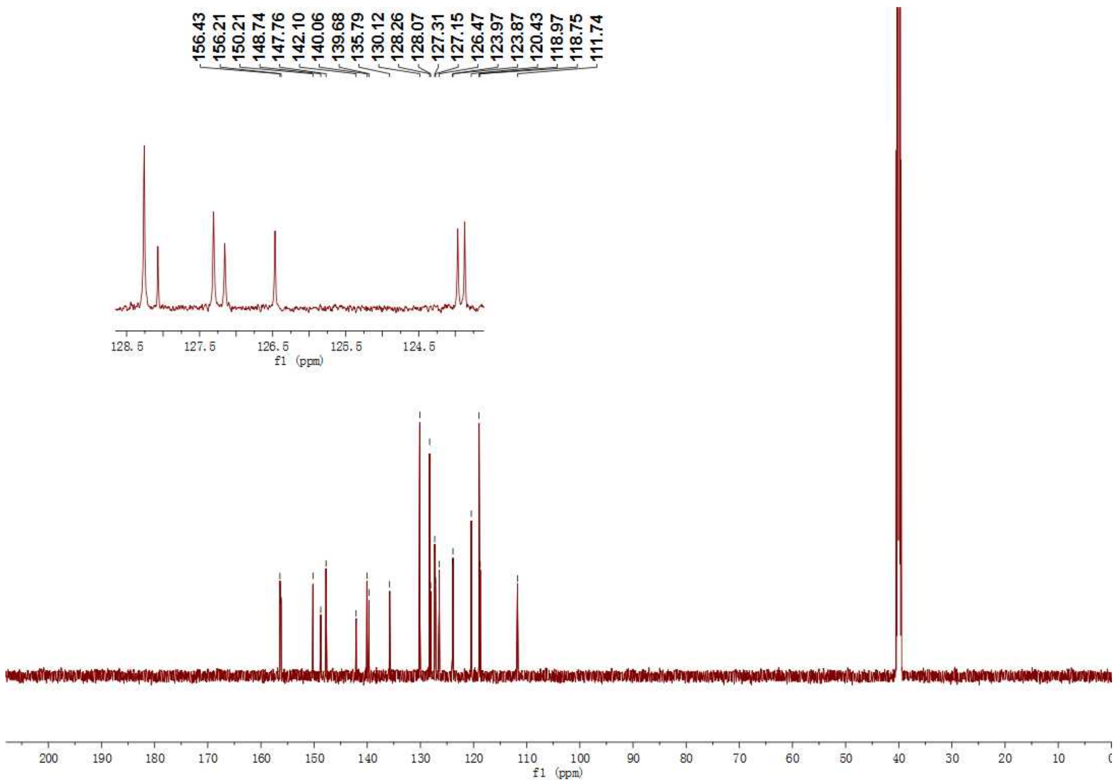

156.43
156.21
150.21
148.74
147.76
142.10
140.06
139.68
135.79
130.12
128.26
128.07
127.31
127.15
126.47
123.97
123.87
120.43
118.97
118.75
111.74
128.5 127.5 126.5 125.5 124.5
f1 (ppm)
200 190 180 170 160 150 140 130 120 110 100 90 80 70 60 50 40 30 20 10
f1 (ppm)

Compd 10g

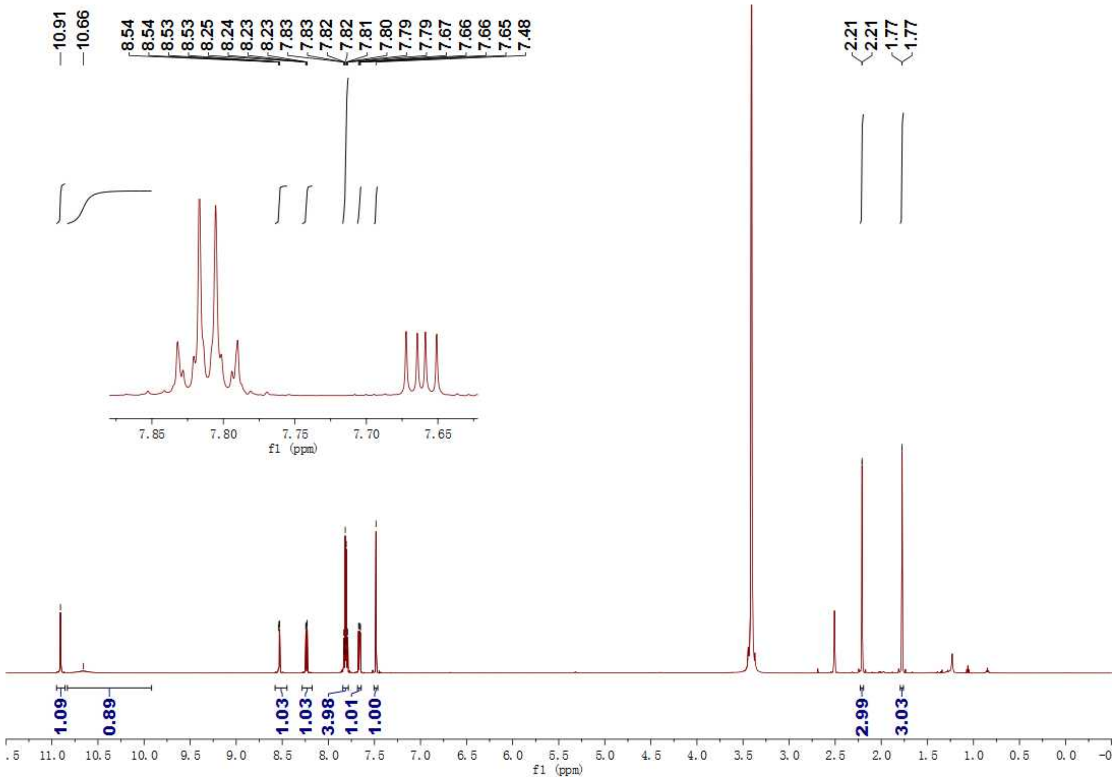
10.91
10.66
8.54
8.54
8.53
8.53
8.25
8.24
8.23
8.23
7.83
7.83
7.82
7.82
7.81
7.80
7.79
7.79
7.67
7.66
7.66
7.65
7.48
2.21
2.21
1.77
1.77
1.09
0.89
1.03
1.03
3.98
1.01
1.00
2.99
3.03
f1 (ppm)

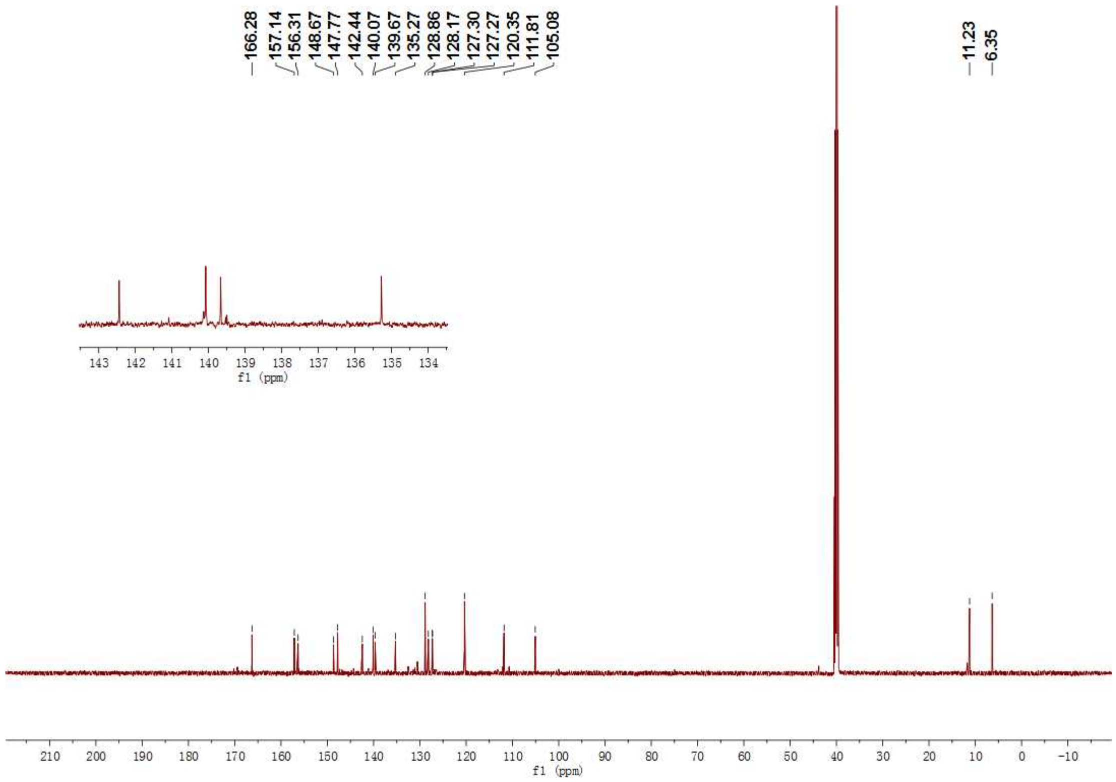
166.28
157.14
156.31
148.67
147.77
142.44
140.07
139.67
135.27
128.86
128.17
127.30
127.27
120.35
111.81
105.08
11.23
6.35
f1 (ppm)

Compd 10h

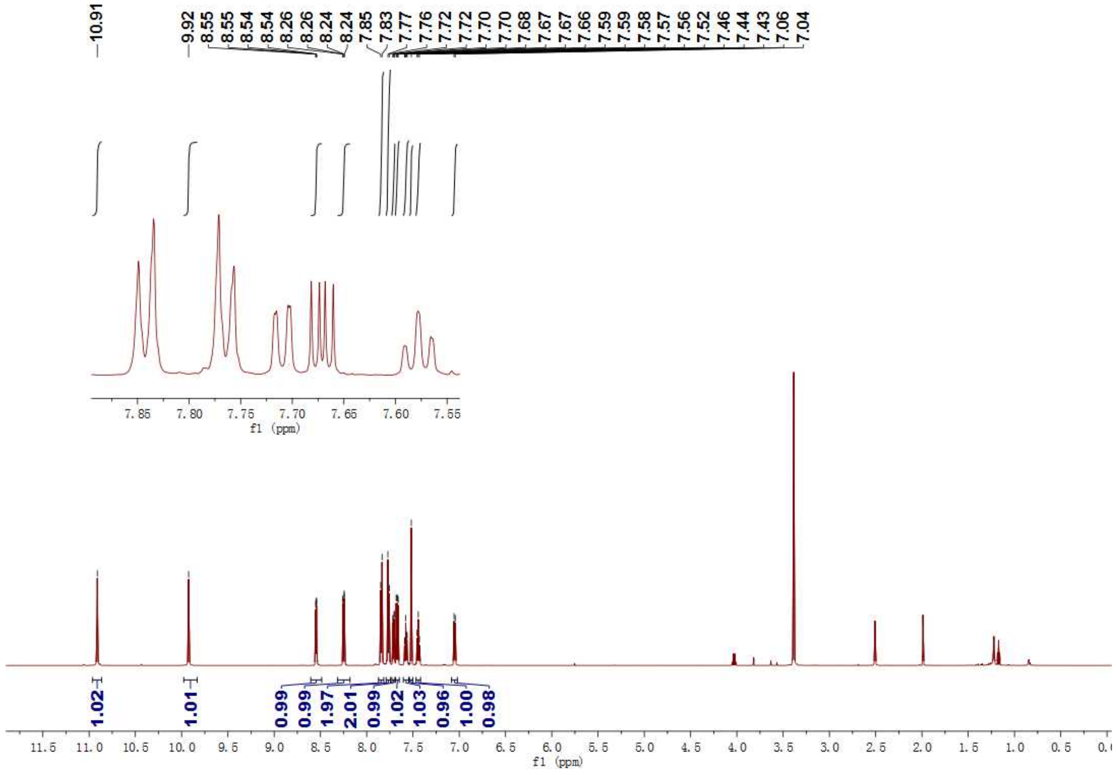

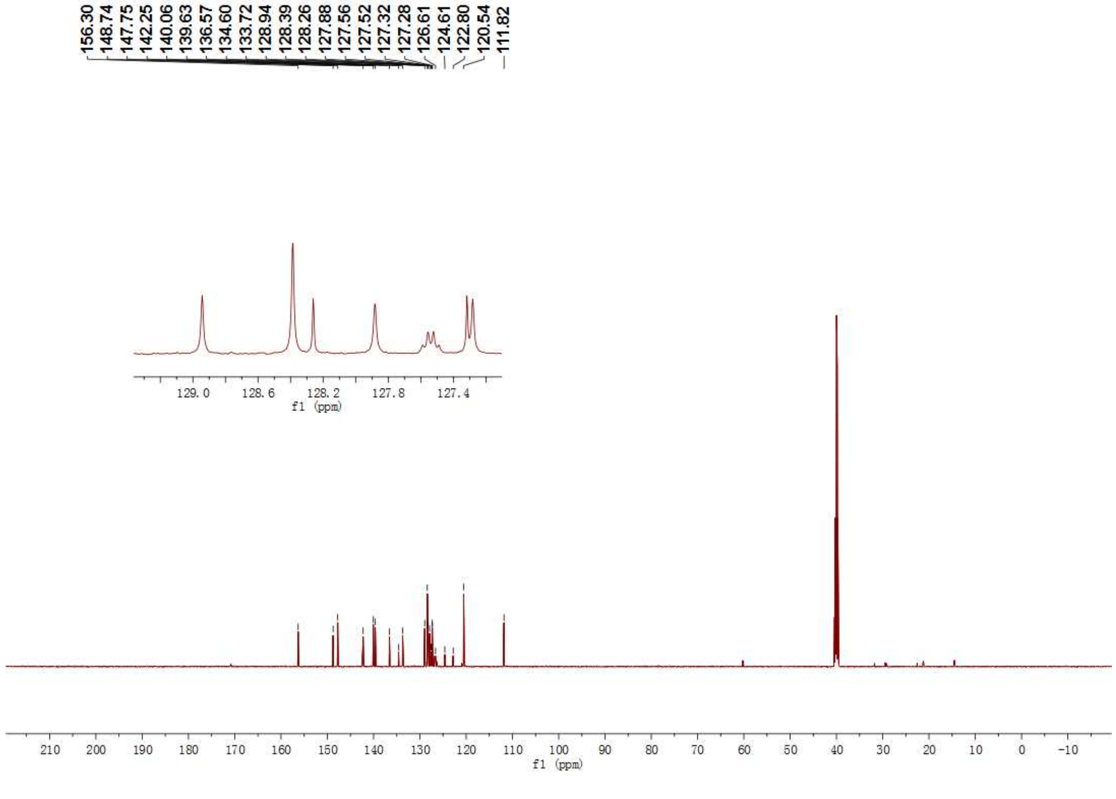

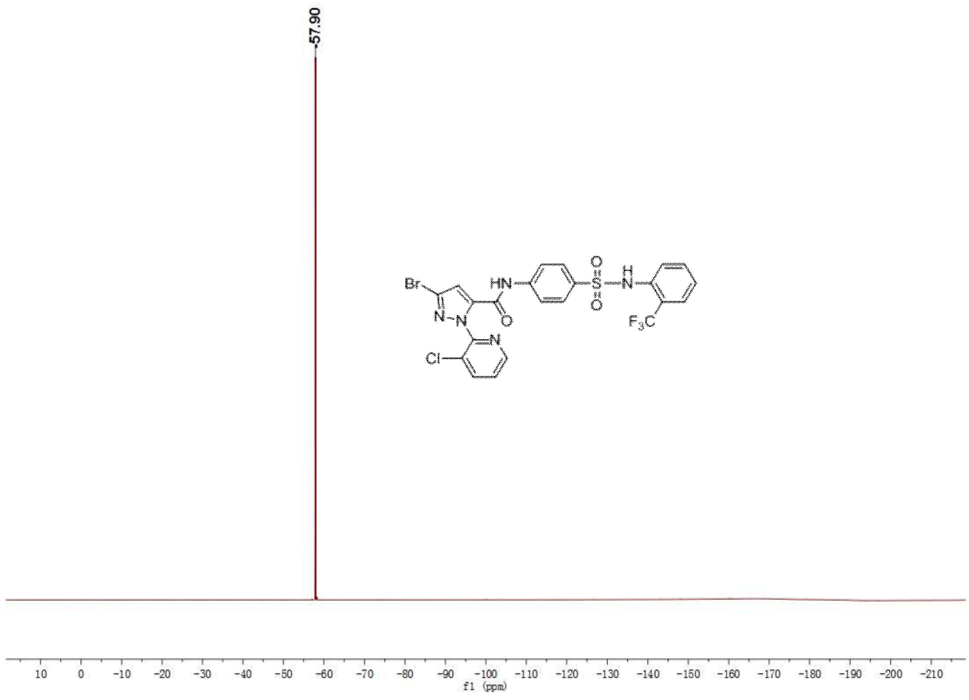
-57.90
f1 (ppm)

Compd 10i

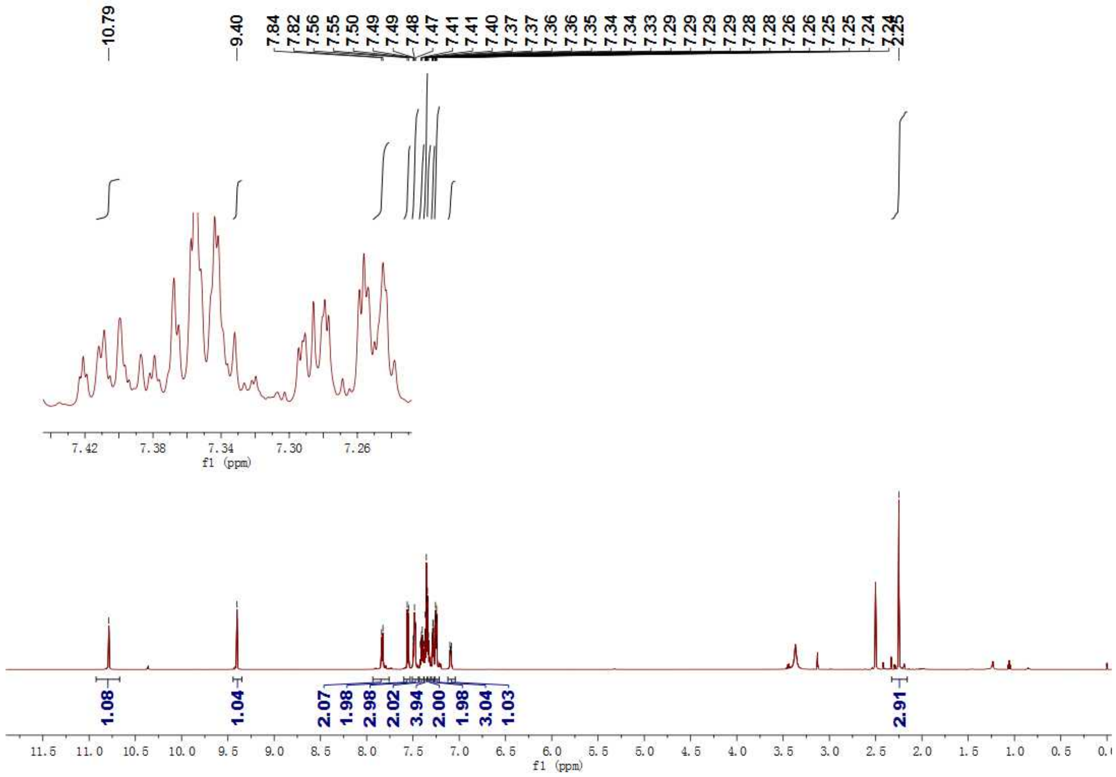
10.79
9.40
7.84 7.82 7.56 7.55 7.50 7.49 7.48 7.47 7.41 7.40 7.37 7.37 7.36 7.35 7.34 7.34 7.33 7.29 7.29 7.29 7.29 7.28 7.26 7.26 7.25 7.25 7.24 7.24
2.25
1.08
1.04
2.07 1.98 2.98 2.02 3.94 2.00 1.98 3.04 1.03
2.91
f1 (ppm)

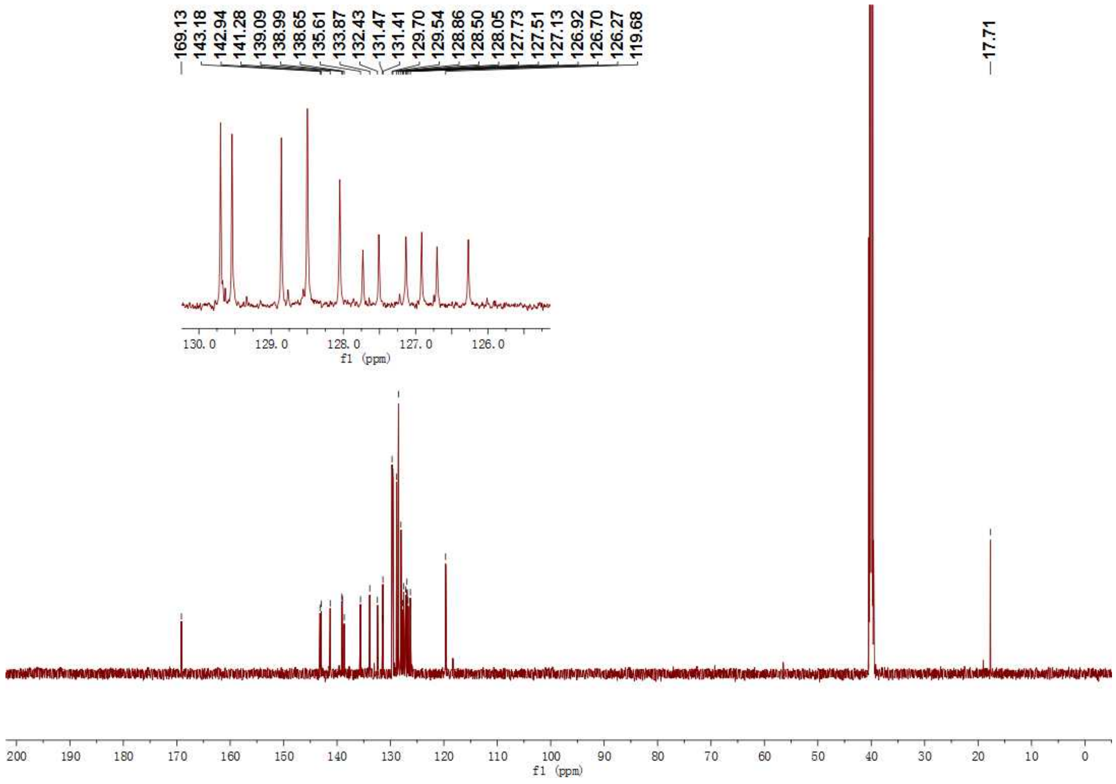
169.13
143.18
142.94
141.28
139.09
138.99
138.65
135.61
133.87
132.43
131.47
131.41
129.70
129.54
128.86
128.50
128.05
127.73
127.51
127.13
126.92
126.70
126.27
119.68
17.71
f1 (ppm)

Compd 10j

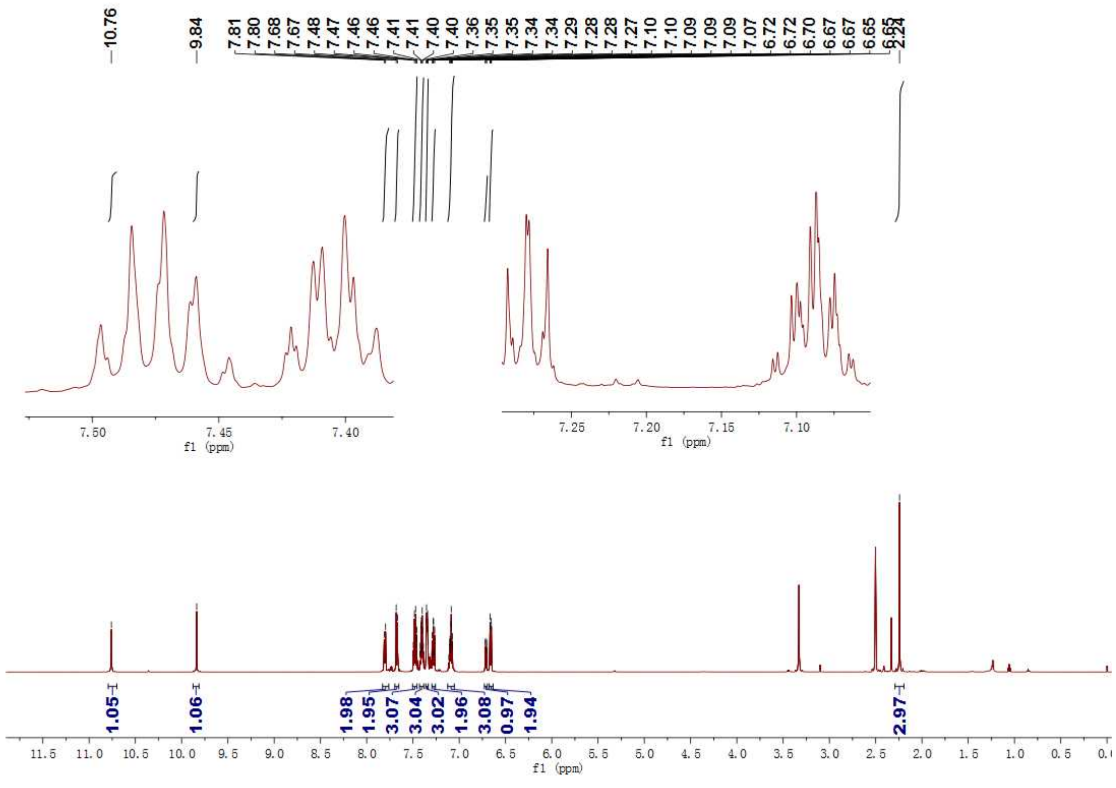
10.76
9.84
7.81
7.80
7.68
7.67
7.48
7.47
7.46
7.46
7.41
7.40
7.40
7.36
7.35
7.35
7.34
7.29
7.28
7.28
7.27
7.10
7.10
7.09
7.09
7.09
7.07
6.72
6.70
6.67
6.65
2.24
1.05
1.06
1.98
1.95
3.07
3.04
3.02
1.96
3.08
0.97
1.94
2.97
f1 (ppm)

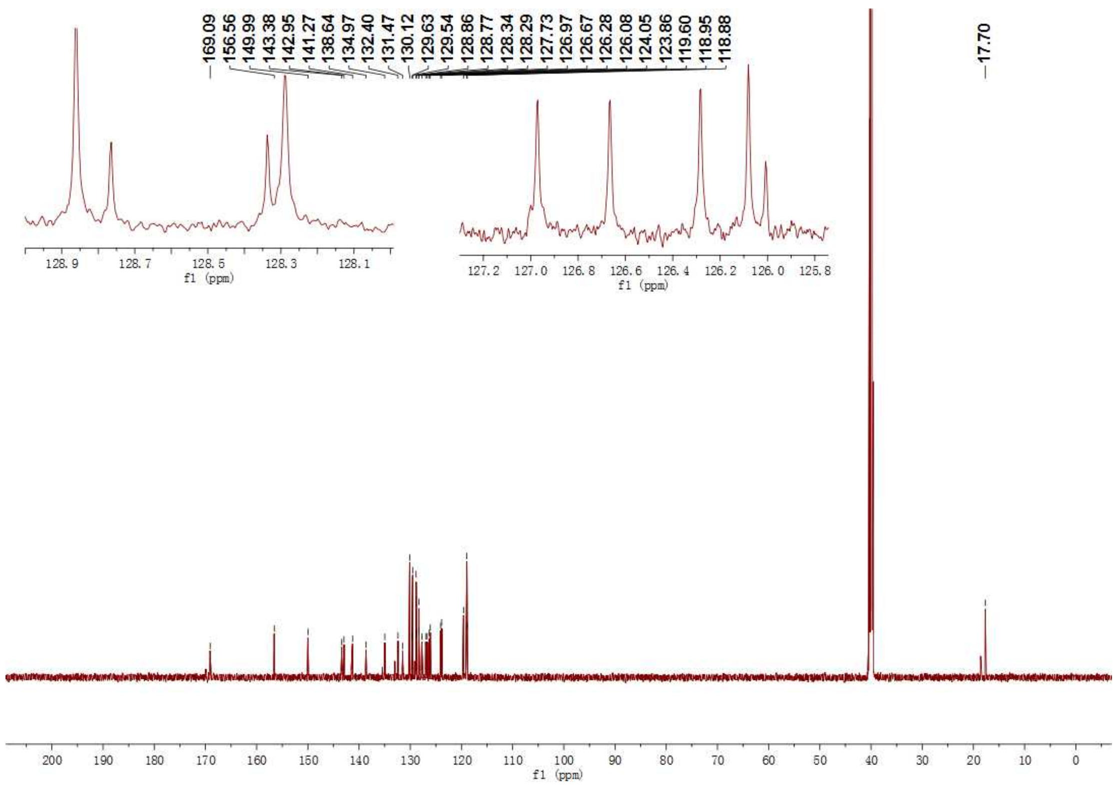
169.09
156.56
149.99
143.38
142.95
141.27
138.64
134.97
132.40
131.47
130.12
129.63
129.54
128.86
128.77
128.34
128.29
127.73
126.97
126.67
126.28
126.08
124.05
123.86
119.60
118.95
118.88
17.70
f1 (ppm)

Compd 10k

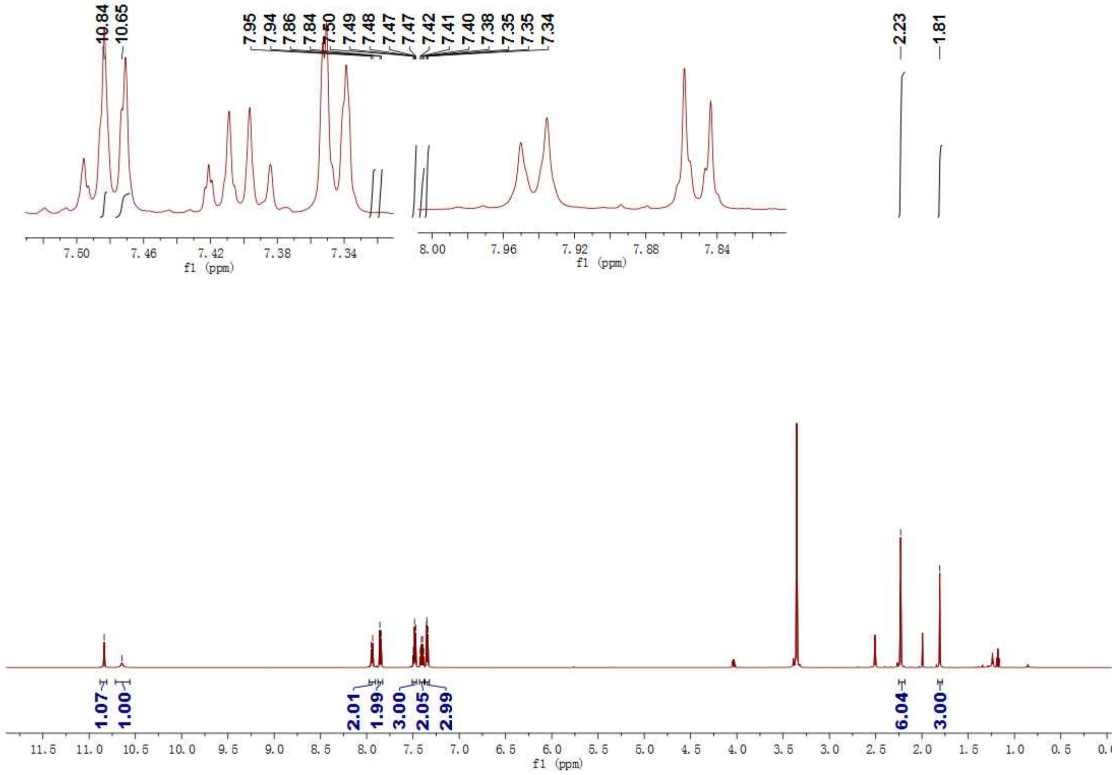
10.84
10.65
7.95
7.94
7.86
7.84
7.50
7.49
7.48
7.47
7.47
7.42
7.41
7.40
7.38
7.35
7.35
7.34
2.23
1.81
1.07
1.00
2.01
1.99
3.00
2.05
2.99
6.04
3.00
f1 (ppm)

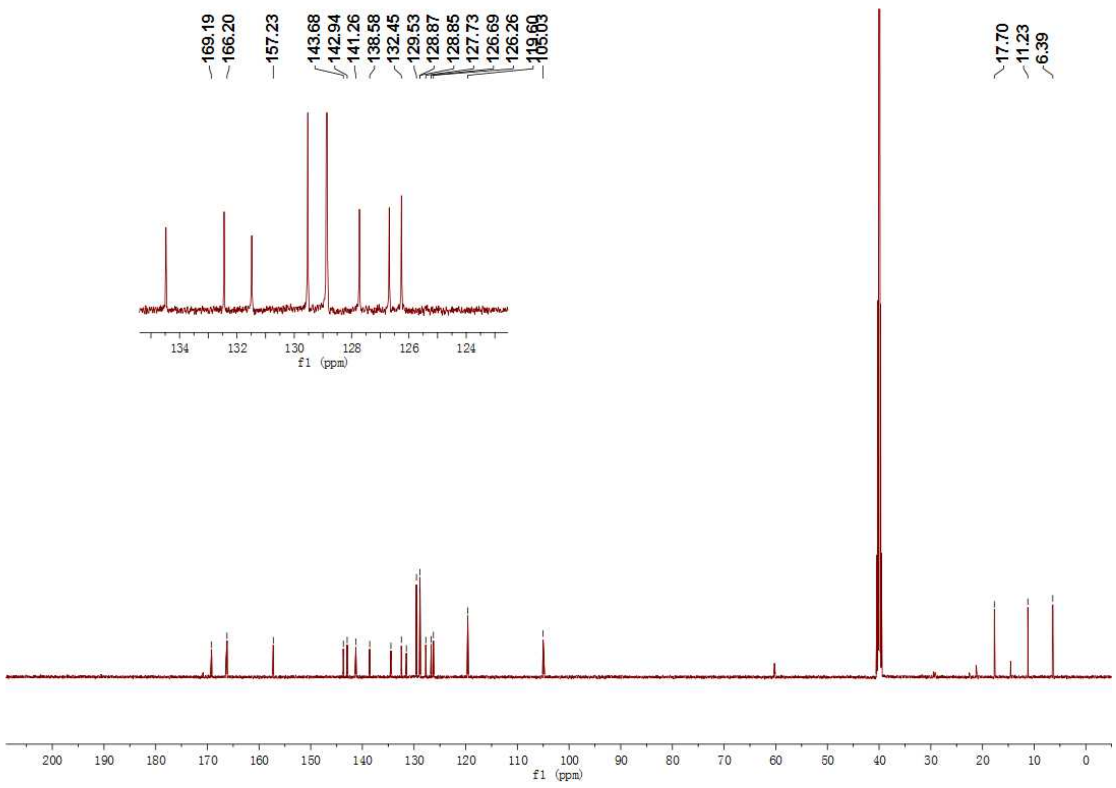

Compd 10l

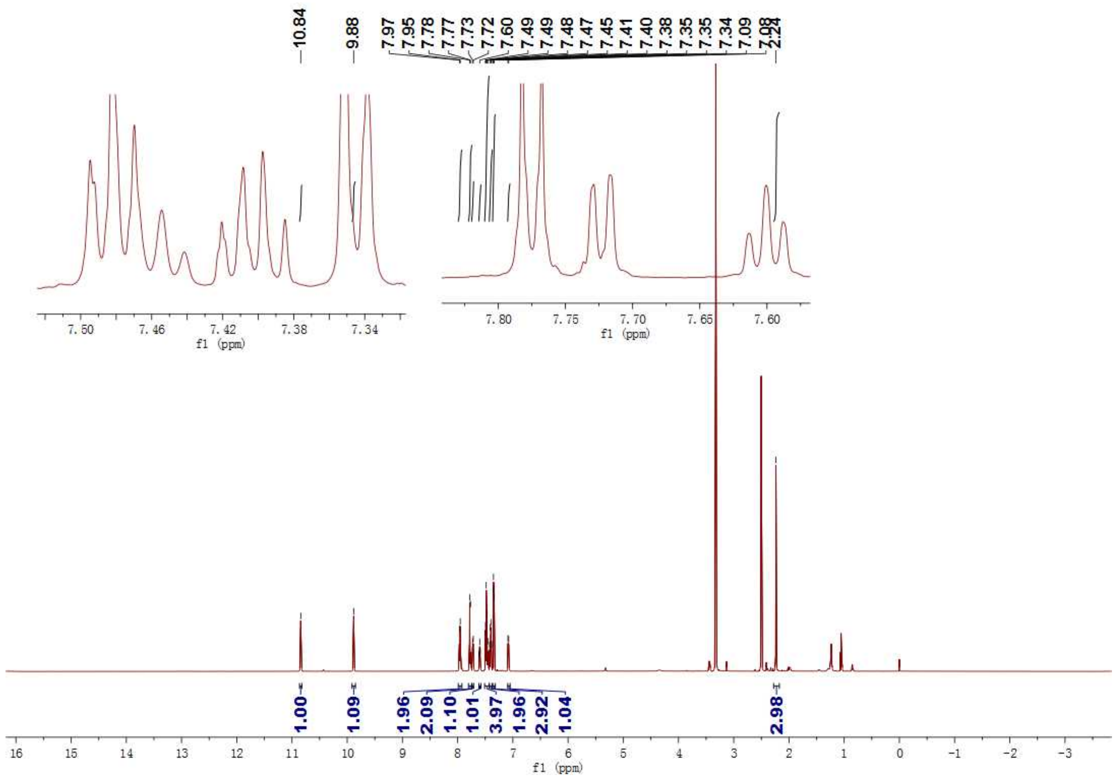

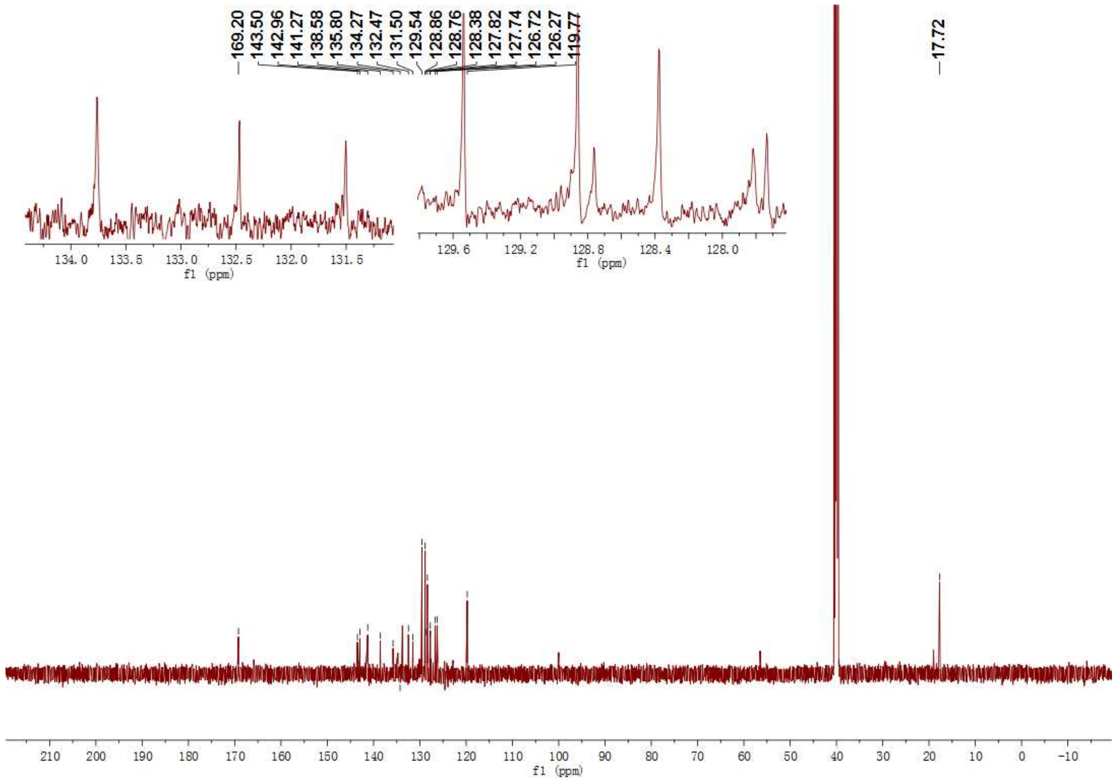
169.20
143.50
142.96
141.27
138.58
135.80
134.27
132.47
131.50
129.54
128.86
128.76
128.38
127.82
127.74
126.72
126.27
119.77
17.72
134.0 133.5 133.0 132.5 132.0 131.5
f1 (ppm)
129.6 129.2 128.8 128.4 128.0
f1 (ppm)
210 200 190 180 170 160 150 140 130 120 110 100 90 80 70 60 50 40 30 20 10 0 -10
f1 (ppm)

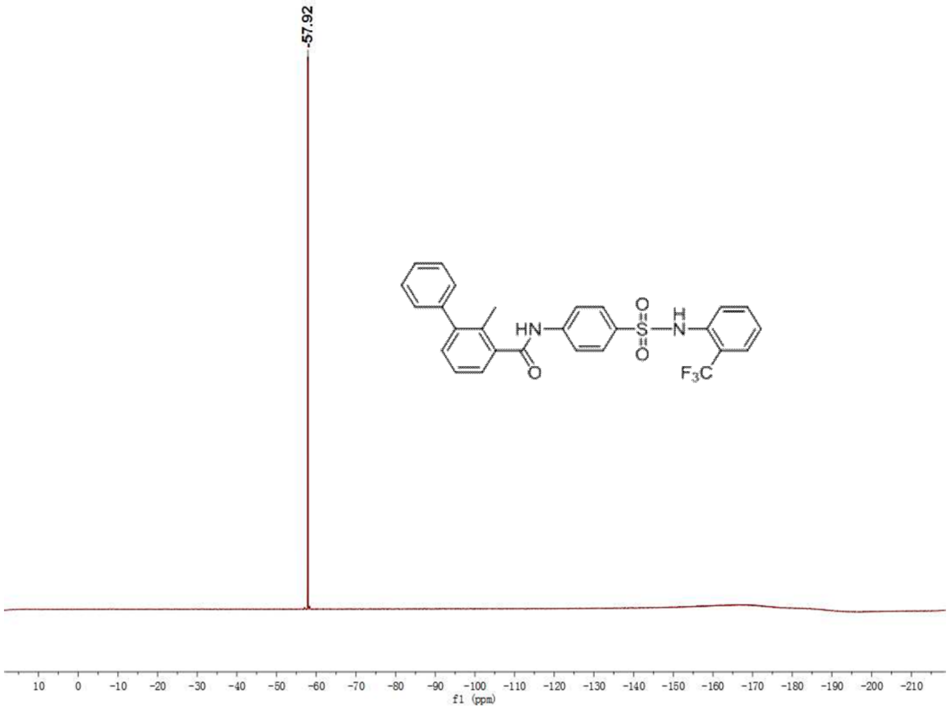
-57.92
HN
H
N
O
S
O
O
F3C
10 0 -10 -20 -30 -40 -50 -60 -70 -80 -90 -100 -110 -120 -130 -140 -150 -160 -170 -180 -190 -200 -210
f1 (ppm)

Compd 10m

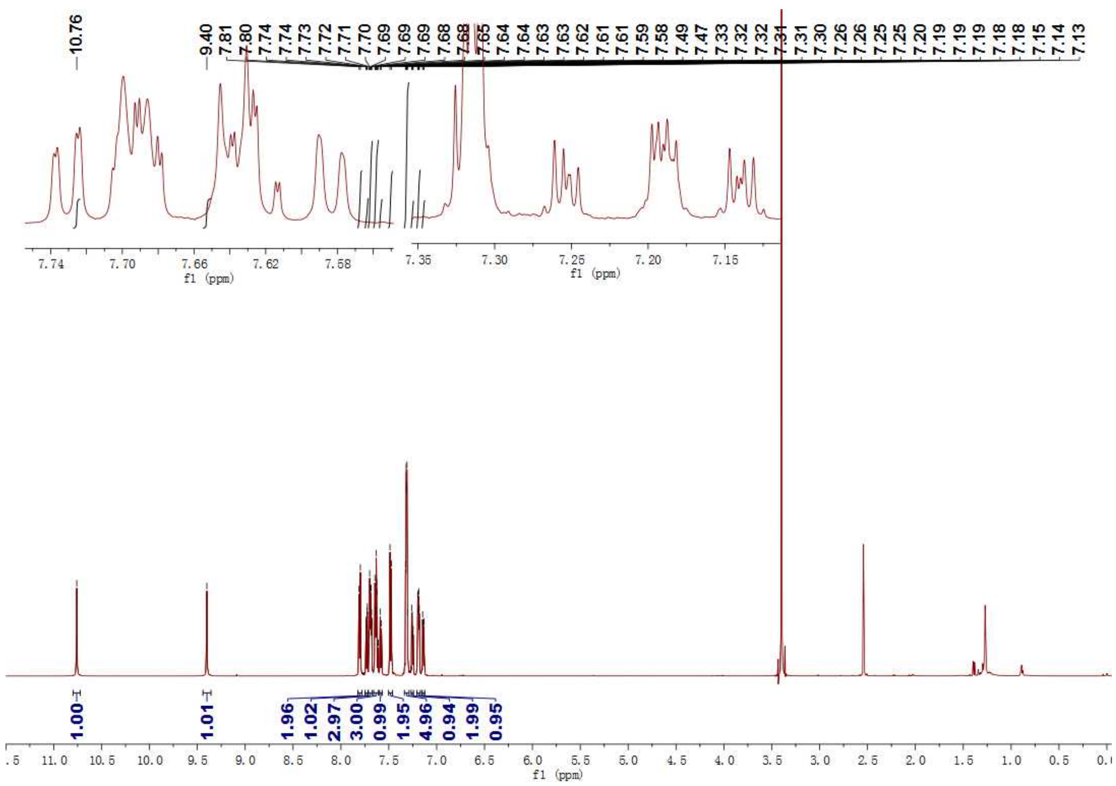

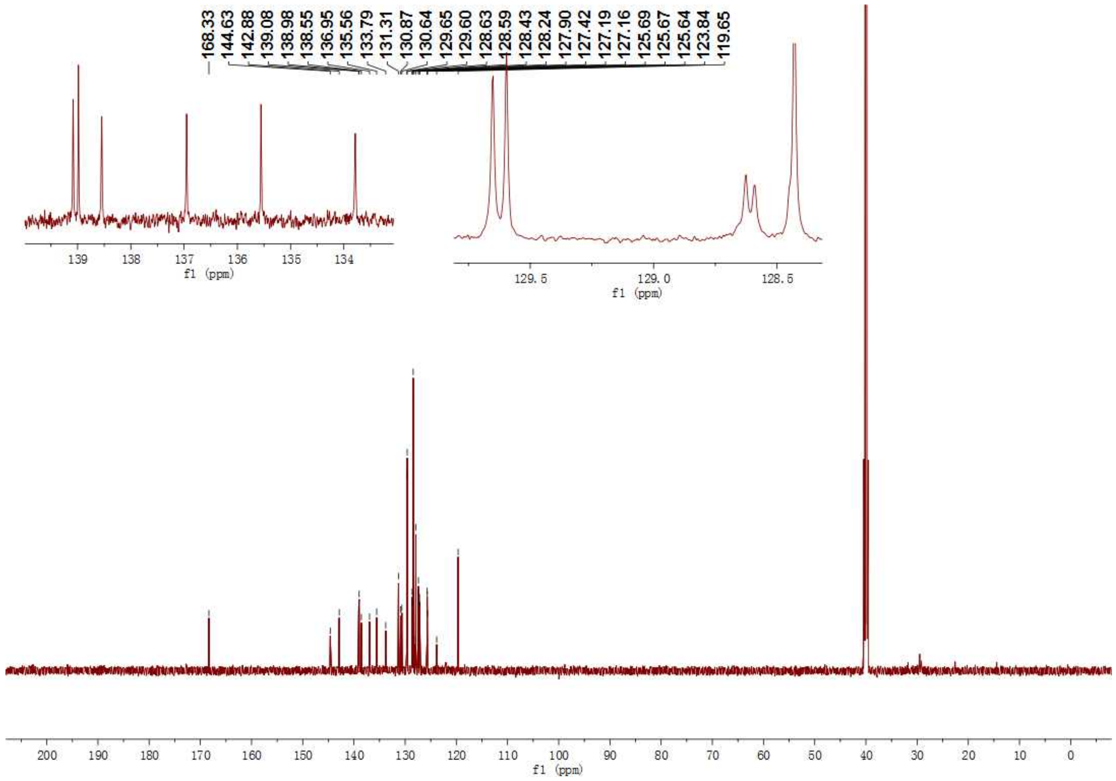

Compd 10n

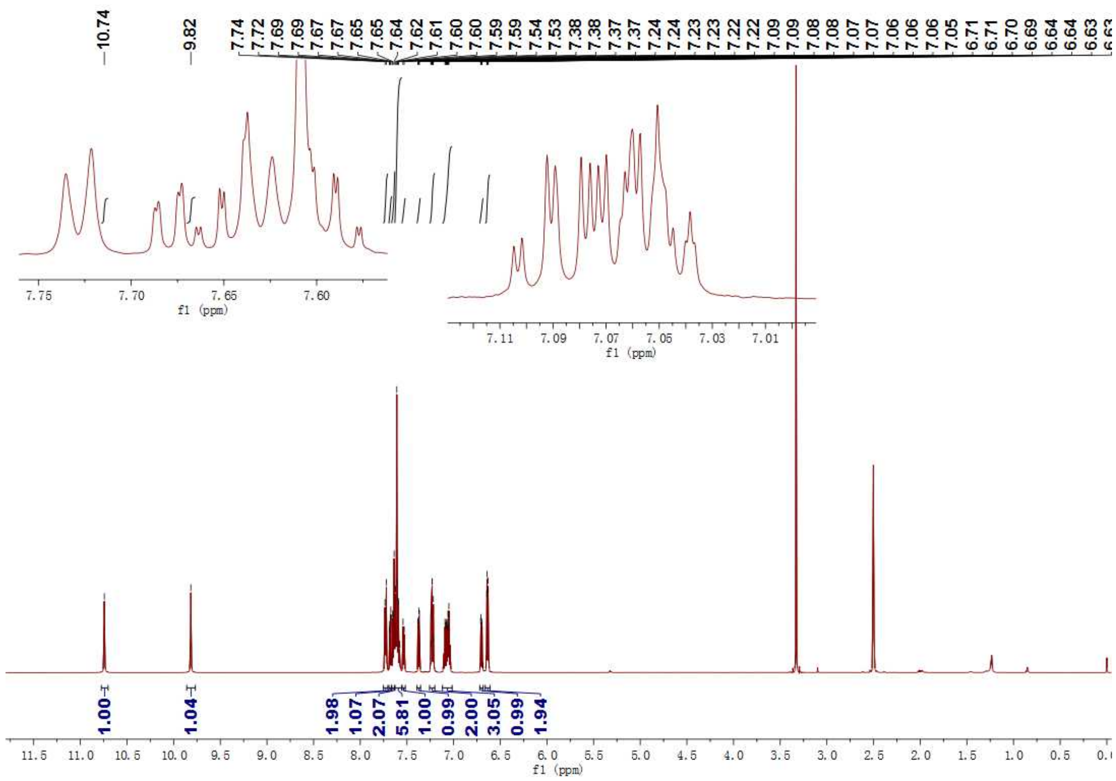
10.74
9.82
7.74 7.72 7.69 7.69 7.67 7.67 7.65 7.65 7.64 7.62 7.61 7.60 7.60 7.59 7.59 7.54 7.53 7.38 7.38 7.37 7.24 7.24 7.23 7.23 7.22 7.22 7.09 7.09 7.08 7.08 7.07 7.07 7.06 7.06 7.06 7.05 6.71 6.70 6.69 6.64 6.63
1.00 1.04 1.98 1.07 2.07 5.81 1.00 0.99 2.00 3.05 0.99 1.94
f1 (ppm)

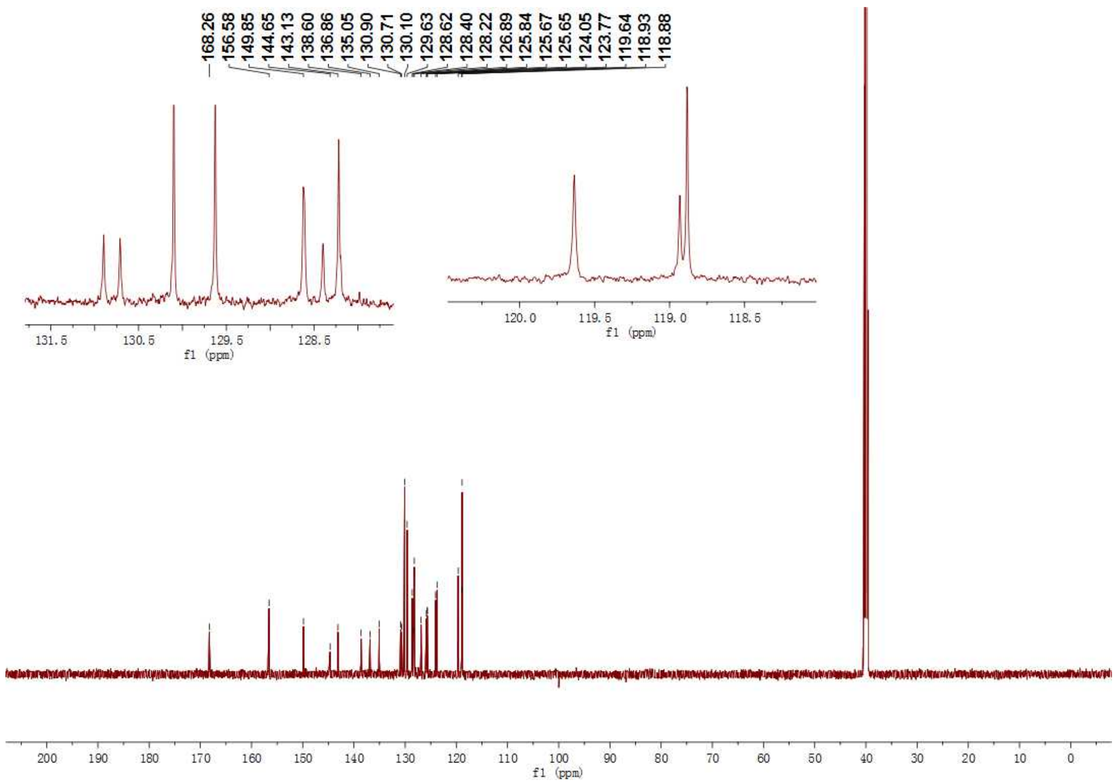
168.26 156.58 149.85 144.65 143.13 138.60 136.86 135.05 130.90 130.71 130.10 129.63 128.62 128.40 128.22 126.89 125.84 125.67 125.65 124.05 123.77 119.64 118.93 118.88
f1 (ppm)

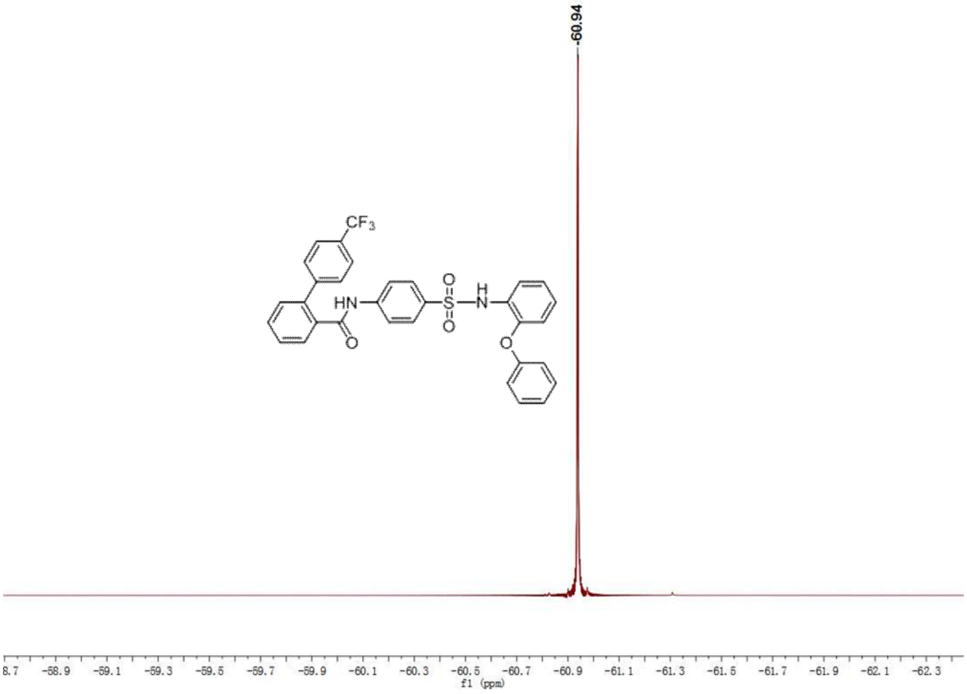

-60.94
CF3
HN
O
S
H
N
O
O
O
f1 (ppm)

Compd 10o

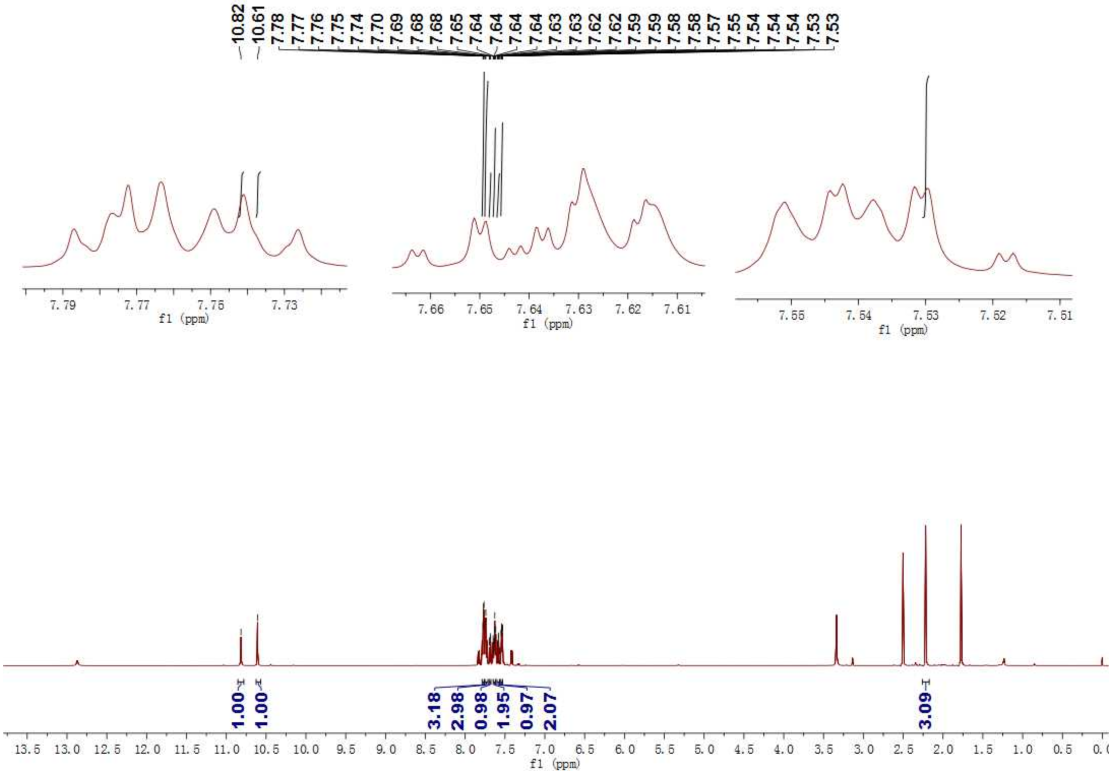

10.82
10.61
7.78 7.77 7.76 7.75 7.74 7.70 7.69 7.68 7.68 7.65 7.64 7.64 7.64 7.64 7.63 7.62 7.62 7.59 7.58 7.58 7.57 7.55 7.54 7.54 7.54 7.53 7.53
1.00
1.00
3.18
2.98
0.98
1.95
0.97
2.07
3.09
f1 (ppm)

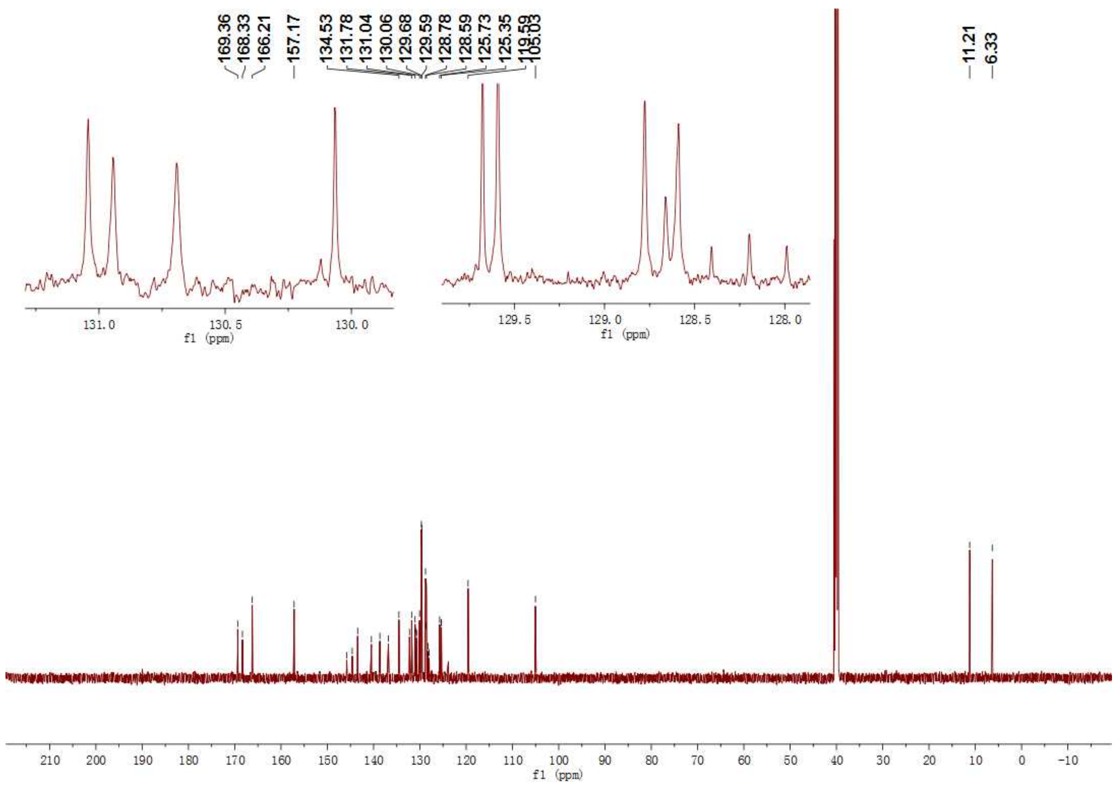
169.36
168.33
166.21
157.17
134.53
131.78
131.04
130.06
129.68
129.59
128.78
128.59
125.73
125.35
119.59
105.03
11.21
6.33
131.0
130.5
130.0
f1 (ppm)
129.5
129.0
128.5
128.0
f1 (ppm)
210 200 190 180 170 160 150 140 130 120 110 100 90 80 70 60 50 40 30 20 10 0 -10
f1 (ppm)

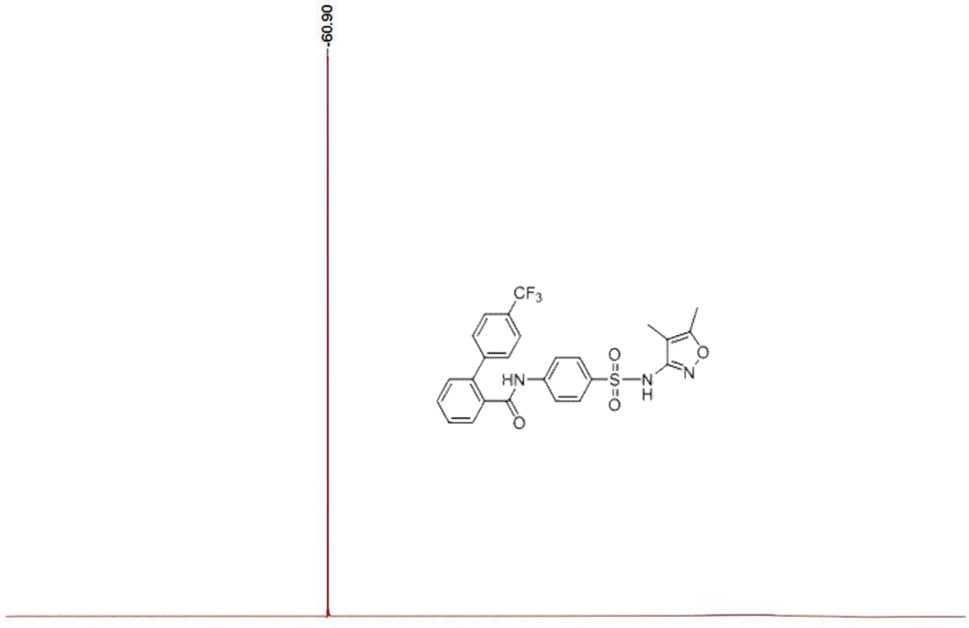
-60.90
CF3
HN
O
S
O
N
H
N
O
O
10 0 -10 -20 -30 -40 -50 -60 -70 -80 -90 -100 -110 -120 -130 -140 -150 -160 -170 -180 -190 -200 -210
f1 (ppm)

Compd 10p

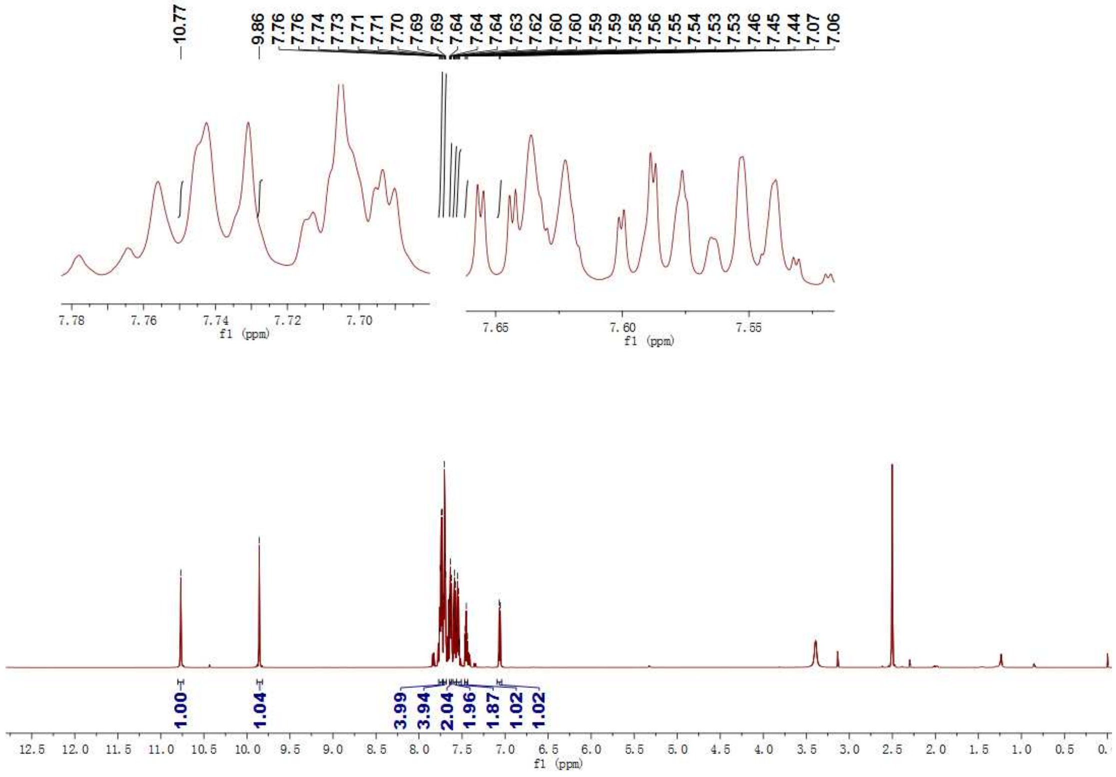
10.77
9.86
7.76
7.76
7.74
7.73
7.71
7.71
7.70
7.69
7.69
7.64
7.64
7.64
7.63
7.62
7.60
7.60
7.59
7.59
7.58
7.56
7.55
7.54
7.53
7.53
7.46
7.45
7.44
7.07
7.06
1.00
1.04
3.99
3.94
2.04
1.96
1.87
1.02
1.02
f1 (ppm)

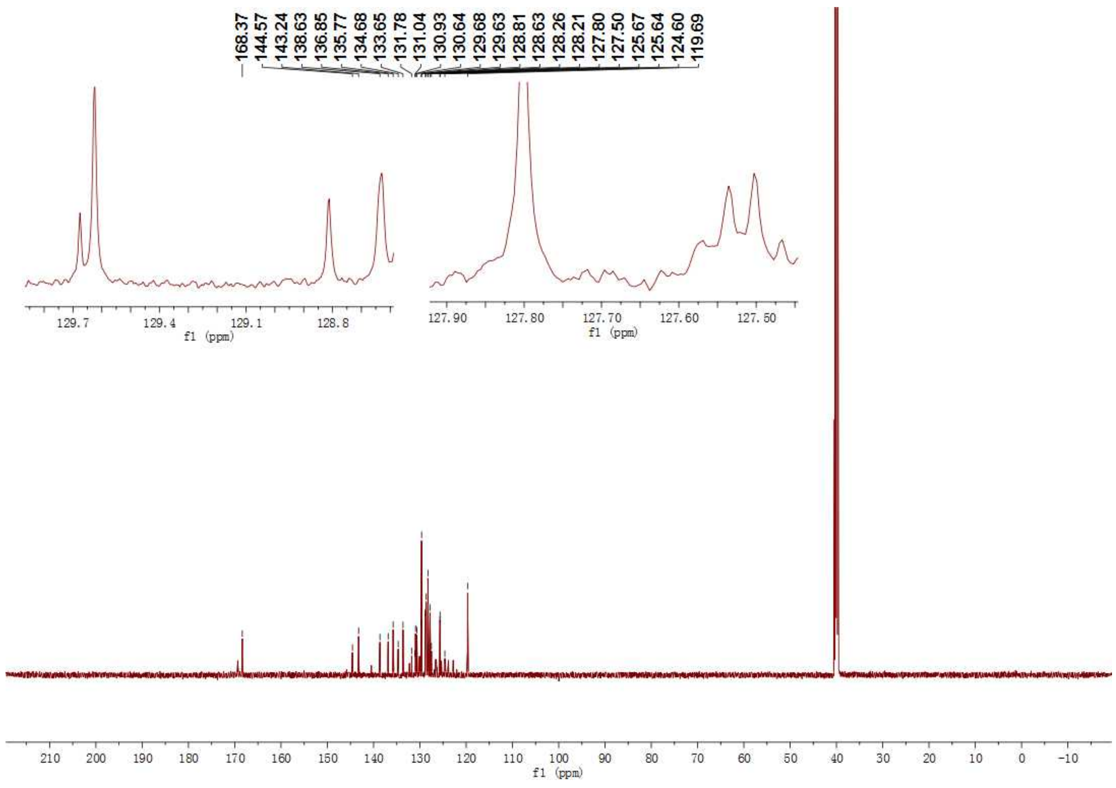
168.37
144.57
143.24
138.63
136.85
135.77
134.68
133.65
131.78
131.04
130.93
130.64
129.68
129.63
128.81
128.63
128.26
128.21
127.80
127.50
125.67
125.64
124.60
119.69
f1 (ppm)

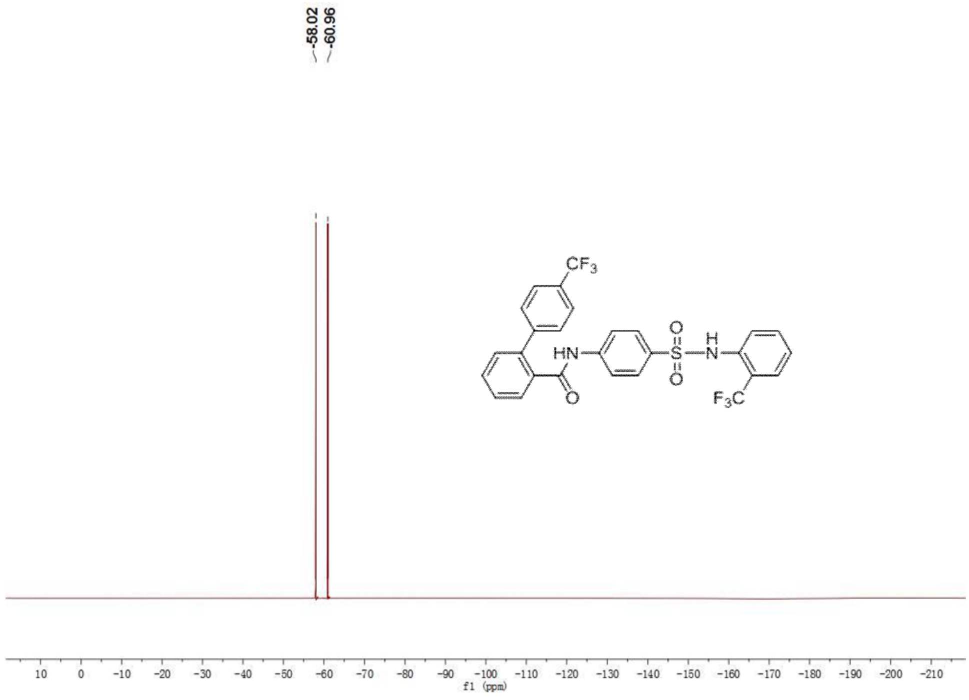
-58.02
-60.96
CF3
HN
O
S
O
H
N
O
F3C
10
0
-10
-20
-30
-40
-50
-60
-70
-80
-90
-100
-110
-120
-130
-140
-150
-160
-170
-180
-190
-200
-210
f1 (ppm)

Compd 10q

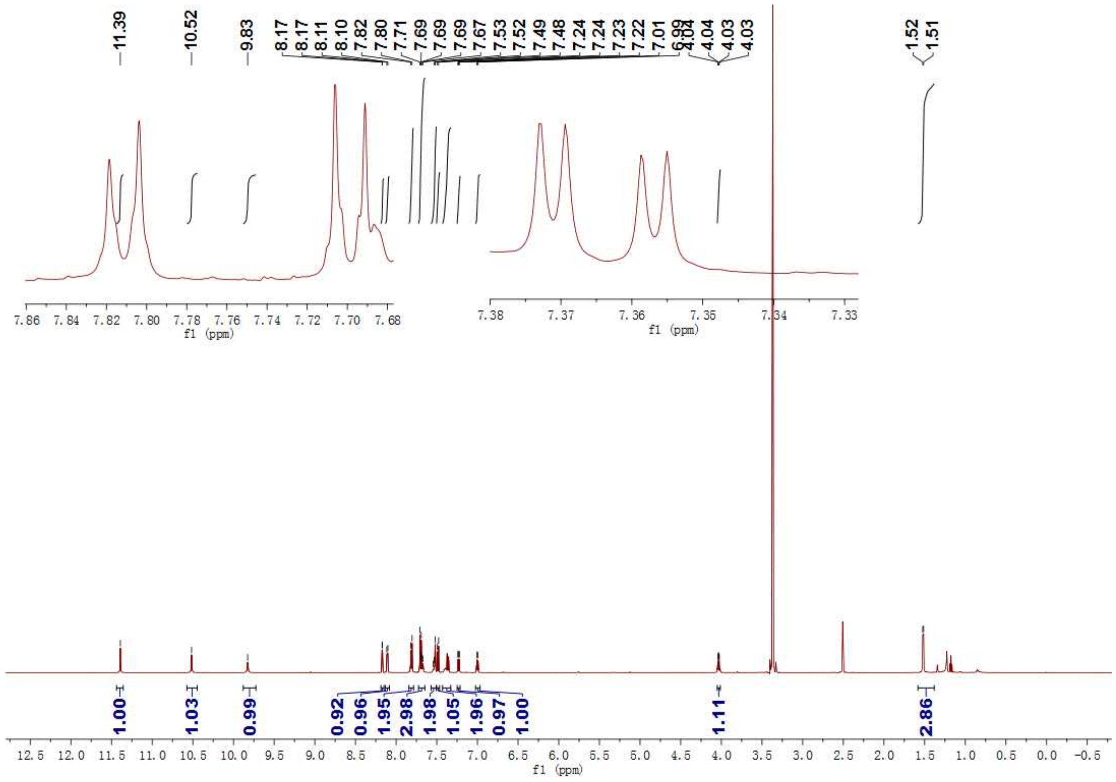
11.39
10.52
9.83
8.17
8.17
8.11
8.10
7.82
7.80
7.71
7.69
7.69
7.69
7.67
7.53
7.52
7.49
7.48
7.24
7.24
7.23
7.22
7.01
6.99
4.04
4.04
4.03
4.03
1.52
1.51
7.86
7.84
7.82
7.80
7.78
7.76
7.74
7.72
7.70
7.68
f1 (ppm)
7.38
7.37
7.36
7.35
7.34
7.33
f1 (ppm)
1.00
1.03
0.99
0.92
0.96
1.95
2.98
1.98
1.05
1.96
0.97
1.00
1.11
2.86
12.5
12.0
11.5
11.0
10.5
10.0
9.5
9.0
8.5
8.0
7.5
7.0
6.5
6.0
5.5
5.0
4.5
4.0
3.5
3.0
2.5
2.0
1.5
1.0
0.5
0.0
-0.5
f1 (ppm)

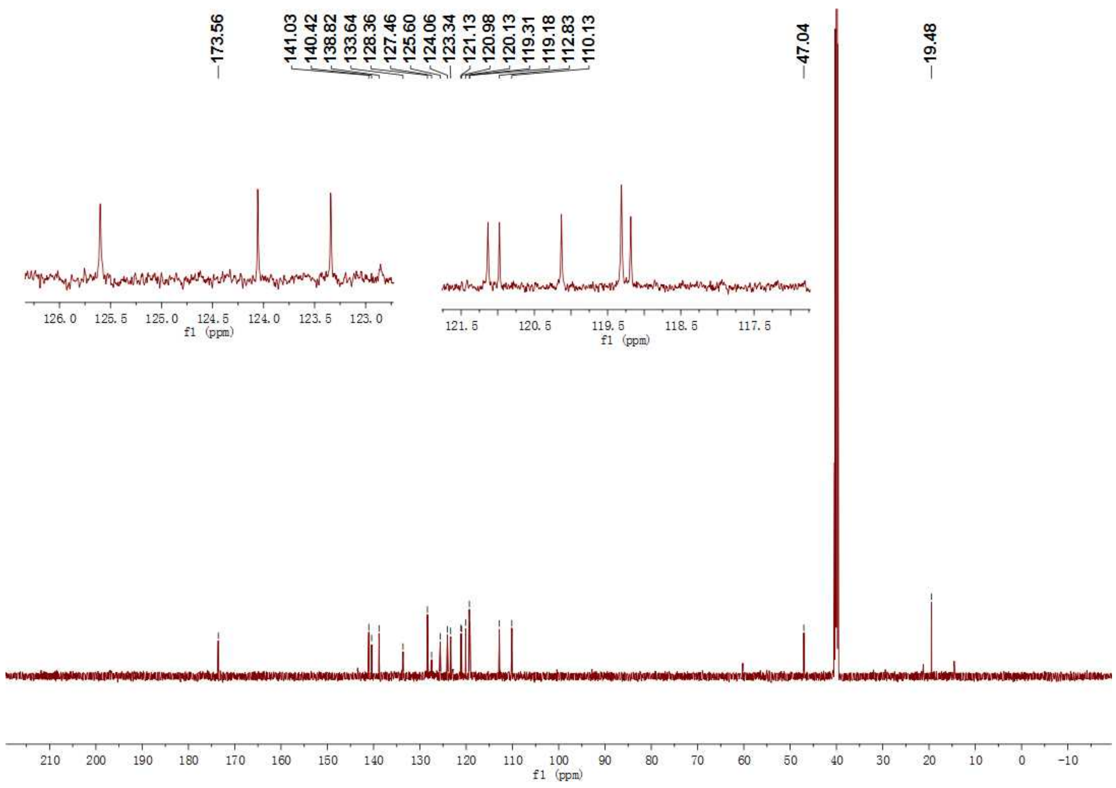
173.56
141.03
140.42
138.82
133.64
128.36
127.46
125.60
124.06
123.34
121.13
120.98
120.13
119.31
119.18
112.83
110.13
47.04
19.48
f1 (ppm)

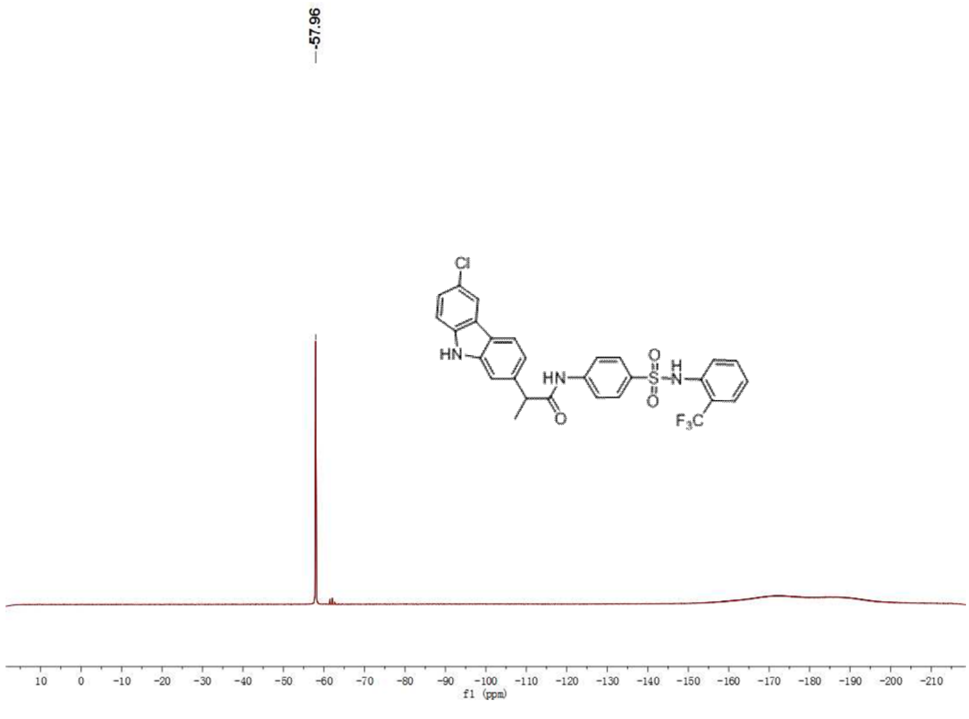
-57.96
Cl
HN
HN
O
S
O
H
N
O
F3C
f1 (ppm)

Compd 10r

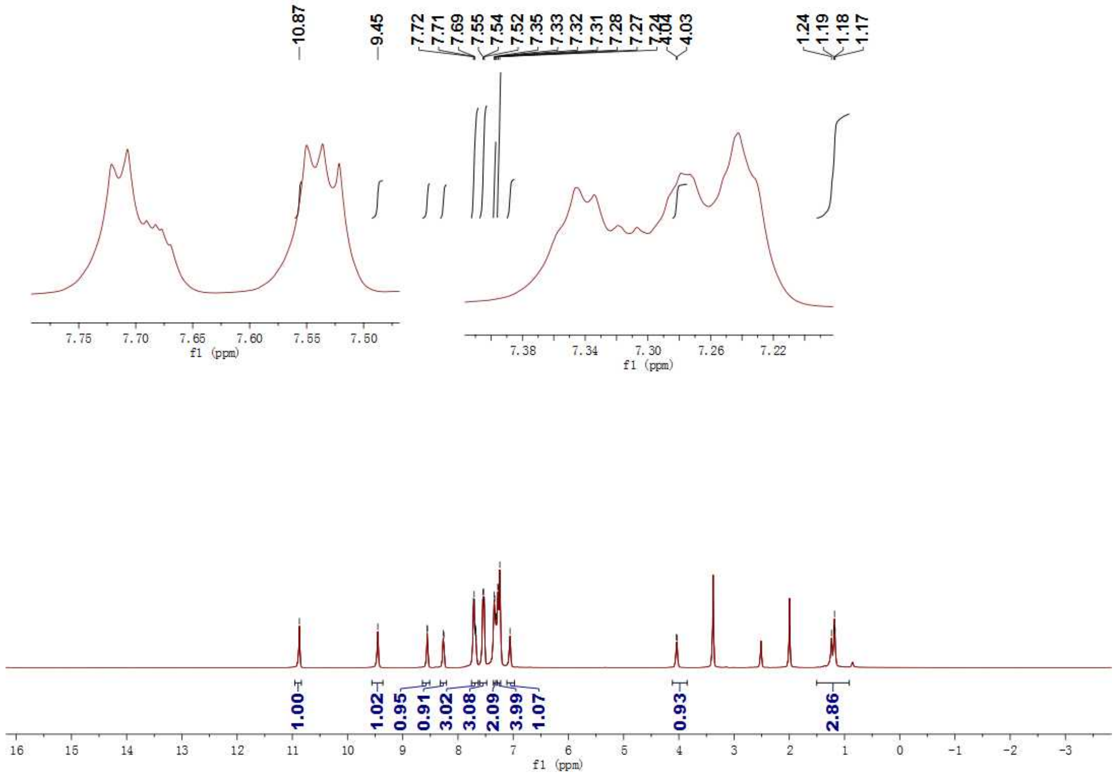
10.87
9.45
7.72
7.71
7.69
7.55
7.54
7.52
7.35
7.33
7.32
7.31
7.28
7.27
7.24
4.04
4.03
1.24
1.19
1.18
1.17
1.00
1.02
0.95
0.91
3.02
3.08
2.09
3.99
1.07
0.93
2.86
f1 (ppm)

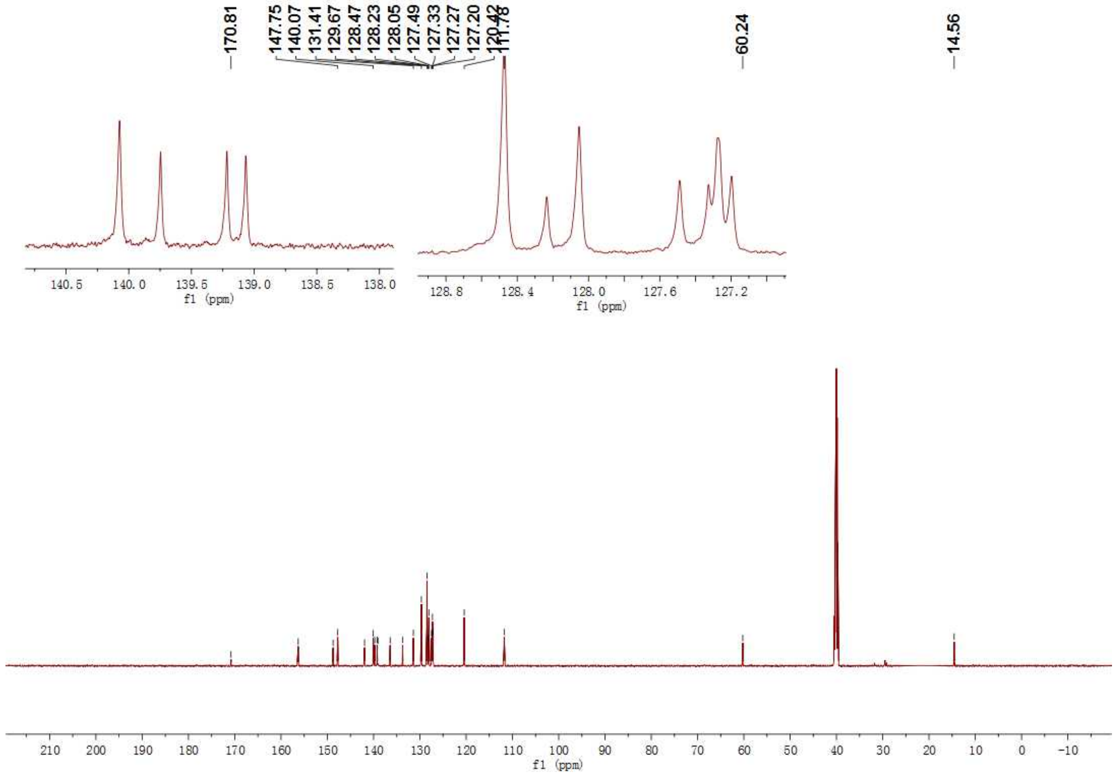
170.81
147.75
140.07
131.41
129.67
128.47
128.23
128.05
127.49
127.33
127.27
127.20
120.42
111.78
60.24
14.56
f1 (ppm)
